# Synthesis, Biological Evaluation, Molecular Docking and Molecular Dynamics of Substituted Thieno[2,3-d]pyrimidine Derivatives as Potential Anti-Alzheimer Agents

**DOI:** 10.3390/ijms27146119

**Published:** 2026-07-08

**Authors:** Asma K. Alshamari, Nourhan Magdy, Ebtesam A. Basiony, Nasser A. Hassan, Odeh A. O. Alshammari, Adel A.-H. Abdel-Rahman, Nuha O. S. Alsaif, Mona Z. Alshammari, Ahmed A. Elrashedy, Allam A. Hassan

**Affiliations:** 1 Department of Chemistry, College of Science, Ha’il University, Ha’il 81451, Saudi Arabia; ak.alshamari@uoh.edu.sa (A.K.A.); odeh.alshammari@uoh.edu.sa (O.A.O.A.); n.alseif@uoh.edu.sa (N.O.S.A.); m.althonaian@uoh.edu.sa (M.Z.A.); 2 Department of Chemistry, Faculty of Science, Menofia University, Shbien El-Kom 32511, Egypt; nourhan_magdy1994@yahoo.com (N.M.); ebtesambasiony@gmail.com (E.A.B.); adelnassar63@yahoo.com (A.A.-H.A.-R.); 3 Department of Photochemistry, Synthetic Unit, Chemical Industries Research Institute, National Research Centre, 33 El Buhouth Street, Cairo P.O. Box 12622, Egypt; 4 Natural and Microbial Chemistry Department, Pharmaceutical and Drug Industries Research Institute, National Research Centre, 33 El Buhouth Street, Cairo P.O. Box 12622, Egypt; ahmedelrashedy45@gmail.com; 5 College of Pharmacy, Al-Farahidi University, Baghdad 10021, Iraq; 6 Department of Chemistry, Faculty of Science, Suez University, Suez 43221, Egypt; allam.hassan@sci.suezuni.edu.eg

**Keywords:** β-enaminonitriles, thieno[2,3-d]pyrimidine, acetylcholinesterase inhibition, butyrylcholinesterase, MM/GBSA, SAR analysis, Alzheimer’s disease, dual inhibitors, molecular docking, molecular dynamics

## Abstract

Thienopyrimidine derivatives are emerging as potent scaffolds for cholinesterase inhibition in Alzheimer’s disease therapy. In this work, a novel series of substituted thieno[2,3-d]pyrimidines was synthesized via Gewald’s reaction, followed by cyclization and functionalization through nucleophilic substitution and hydrazone formation. Structural confirmation was achieved using spectroscopic techniques, and biological evaluation was performed against acetylcholinesterase (AChE) and butyrylcholinesterase (BChE), with donepezil and rivastigmine as reference drugs. Compound 4 emerged as the most potent and selective AChE inhibitor (IC_50_ = 0.58 µM), while compound **7** also showed strong AChE inhibition (IC_50_ = 0.63 µM). Notably, compound **9** exhibited superior BChE inhibition (IC_50_ = 3.05 µM) compared to donepezil (IC_50_ = 8.41 µM). Dual inhibitory activity was observed for compounds **5**, **6**, and **11**, highlighting their multitarget potential. Molecular dynamics simulations (200 ns) and MM/GBSA binding free energy calculations provided mechanistic insights. Compound **4** showed the most favorable binding energy (ΔGbind = −59.16 kcal/mol), driven by hydrogen bonds with Tyr121 and Glu199 and π-π stacking with Trp83. Residue-level decomposition identified Tyr121, Trp83, Glu199, and Tyr338 as critical contributors to binding stability. Structure–activity relationship analysis confirmed that nitrogen-containing substituents and cyclic amino moieties enhance potency, whereas bulky aromatic groups reduce activity. These findings establish thieno[2,3-d]pyrimidine derivatives as promising candidates for the development of next-generation anti-Alzheimer agents.

## 1. Introduction

Alzheimer’s disease (AD) is a chronic and progressive neurodegenerative disorder characterized by hallmark pathological features including neurofibrillary tangles [1], deposition of amyloid β_1−42_ peptides [2], and increased oxidative stress together with sustained neuroinflammatory processes, both of which exacerbate neuronal damage and accelerate disease progression [3,4]. Oxidative stress contributes to lipid peroxidation, protein oxidation, and DNA damage, while neuroinflammation mediated by activated microglia and astrocytes amplifies synaptic dysfunction and neuronal loss, forming a vicious cycle that further exacerbates AD pathology [5,6]. These interconnected pathological mechanisms highlight the multifactorial nature of AD and support the development of therapeutic strategies capable of targeting multiple disease-associated pathways.

Affecting millions of people worldwide, AD remains the leading cause of dementia and represents one of the greatest healthcare challenges associated with aging populations [7,8,9,10,11]. Although its etiology remains incompletely understood, several pathological mechanisms have been implicated, including cholinergic dysfunction resulting from reduced acetylcholine (ACh) levels [12], extracellular deposition of amyloid-β plaques [2], and intracellular tau protein aggregation [1]. Acetylcholine degradation by cholinesterase enzymes impairs cholinergic neurotransmission, resulting in deficits in learning, attention, and memory. Consequently, cholinesterase inhibitors remain the cornerstone of symptomatic treatment by increasing synaptic ACh levels. Although these therapies provide meaningful symptomatic benefits for many patients, they neither halt nor reverse disease progression. Moreover, their clinical utility is frequently limited by modest efficacy, adverse effects, poor long-term adherence, and drug–drug interactions, particularly in elderly patients with multiple comorbidities [13,14,15,16,17]. These limitations underscore the urgent need to develop safer and more effective therapeutic agents capable of addressing the complex pathology of AD.

The cholinergic hypothesis remains one of the most extensively validated therapeutic concepts in AD. Reduced acetylcholine (ACh) levels resulting from degeneration of cholinergic neurons contribute directly to deficits in learning, memory, and cognitive function [12]. Accordingly, restoration of cholinergic neurotransmission through inhibition of acetylcholinesterase (AChE) remains the principal pharmacological strategy for symptomatic management of AD [13,14,15,16,17]. Increasing evidence, however, indicates that acetylcholinesterase (AChE) and butyrylcholinesterase (BChE) play distinct roles during disease progression [18]. While AChE predominates in the healthy brain and during the early stages of AD, BChE activity gradually increases as the disease advances, partially compensating for the decline in AChE activity [19,20]. Consequently, dual inhibition of AChE and BChE has emerged as an attractive strategy for maintaining cholinergic neurotransmission throughout different stages of AD.

Donepezil, one of the most widely prescribed drugs for Alzheimer’s disease, was selected as the reference compound in the present study because of its well-established clinical efficacy and reversible inhibition of cholinesterase enzymes [21]. In clinical practice, donepezil is frequently administered alone or in combination with memantine, an N-methyl-D-aspartate (NMDA) receptor antagonist that reduces glutamate-mediated excitotoxicity [22]. Although these therapies improve cognitive symptoms, they do not halt the underlying neurodegenerative processes responsible for disease progression. Consequently, considerable research efforts have focused on developing novel cholinesterase inhibitors with improved potency, selectivity, and additional neuroprotective properties. Accordingly, the thieno[2,3-d]pyrimidine derivatives synthesized in the present study were designed as potential dual cholinesterase inhibitors to identify new lead compounds for the treatment of Alzheimer’s disease [23].

Beyond inhibitory potency, the mechanism and reversibility of enzyme inhibition are critical determinants of therapeutic efficacy and safety. For example, donepezil acts as a reversible inhibitor of cholinesterases, whereas rivastigmine inhibits AChE through a pseudo-irreversible, non-competitive mechanism [15]. Accordingly, characterization of inhibition kinetics has become an essential component in the rational development of novel cholinesterase inhibitors. Although multitarget-directed ligands (MTDLs) have attracted increasing attention for the treatment of Alzheimer’s disease [24,25], dual AChE/BChE inhibition remains a practical and well-established strategy that facilitates structure–activity relationship (SAR) analysis and the identification of promising lead compounds [26].

**Figure 1:**
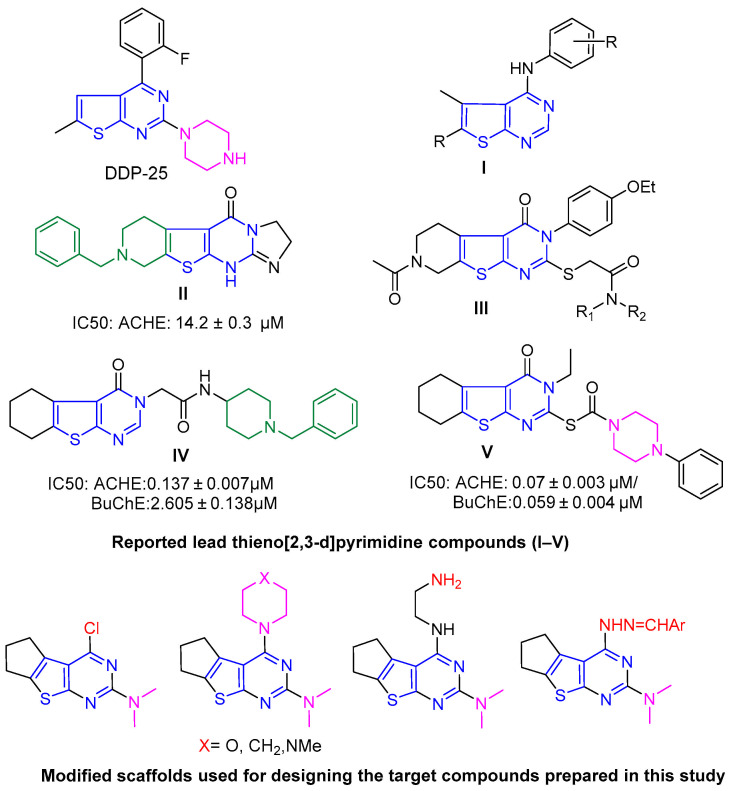
Reported lead thieno[2,3-d]pyrimidines (**I**–**V**) compared with the modified scaffolds designed in this study.

Thieno[2,3-d]pyrimidines have emerged as privileged scaffolds in medicinal chemistry owing to their fused heterocyclic structure and bioisosteric resemblance to adenine [27]. Their synthetic accessibility, structural diversity, and broad spectrum of biological activities—including anti-inflammatory, antioxidant, antineoplastic, and tyrosine kinase inhibitory properties—make them attractive scaffolds for drug discovery [28,29,30,31,32,33]. In the context of Alzheimer’s disease, their antioxidant properties may further contribute to neuroprotection [32,33]. One notable example is DDP-225 (Figure 1), a thieno[2,3-d]pyrimidine derivative developed by Mitsubishi Tanabe Pharmaceuticals to manage attentional deficits associated with cholinergic dysfunction [34]. In addition, thieno[2,3-d]pyrimidines I (Figure 1) have exhibited antioxidant and radical-scavenging activities [32,33], while sugar-conjugated thienopyrimidinone derivatives (compound II, Chart 1) demonstrated promising antioxidant and neuroprotective effects [35]. More recently, tetracyclic thienopyrimidines III (Figure 1) exhibited mixed-type acetylcholinesterase inhibition together with protection against Aβ-induced cytotoxicity and improved memory in Alzheimer’s disease animal models [36]. Furthermore, compound V, reported by Kassab et al. [37], demonstrated potent dual cholinesterase inhibition with lower cytotoxicity than donepezil.

More recently, multifunctional thienopyrimidine-based molecules have further expanded the therapeutic potential of this privileged scaffold. Sharma et al. [38] reported N-benzylpiperidine-oxadiazole hybrids as promising multitarget anti-Alzheimer agents, while Mehta et al. [39] highlighted the evolution of cholinesterase inhibitors toward multifunctional ligands capable of modulating multiple disease pathways simultaneously. Furthermore, Fritzemeier et al. [40] demonstrated that thienopyrimidinone derivatives act as GluN2B/C/D-biased modulators of NMDA receptors, suggesting broader applications of this scaffold in central nervous system drug discovery. Collectively, these studies establish thieno[2,3-d]pyrimidines as versatile and pharmacologically valuable scaffolds suitable for further optimization in Alzheimer’s disease drug discovery.

Building on our previous work demonstrating the synthetic versatility and biological potential of thieno[2,3-d]pyrimidine derivatives [41,42,43,44,45,46,47], the present study explores the systematic structural modification of the thieno[2,3-d]pyrimidine scaffold to identify novel cholinesterase inhibitors for the treatment of Alzheimer’s disease. The target compounds were synthesized from β-enaminonitrile and Viehe’s salt, structurally characterized using spectroscopic techniques, and evaluated for their inhibitory activities against both AChE and BChE. Molecular docking, 200 ns molecular dynamics simulations, and MM/GBSA binding free-energy calculations were performed to elucidate ligand–protein interactions, assess binding stability, and rationalize the observed structure–activity relationships. Accordingly, this integrated experimental and computational approach aims to identify promising lead compounds for the future development of dual cholinesterase inhibitors against Alzheimer’s disease.

## 2. Results and Discussion

### 2.1. Synthesis

**Scheme 1:**
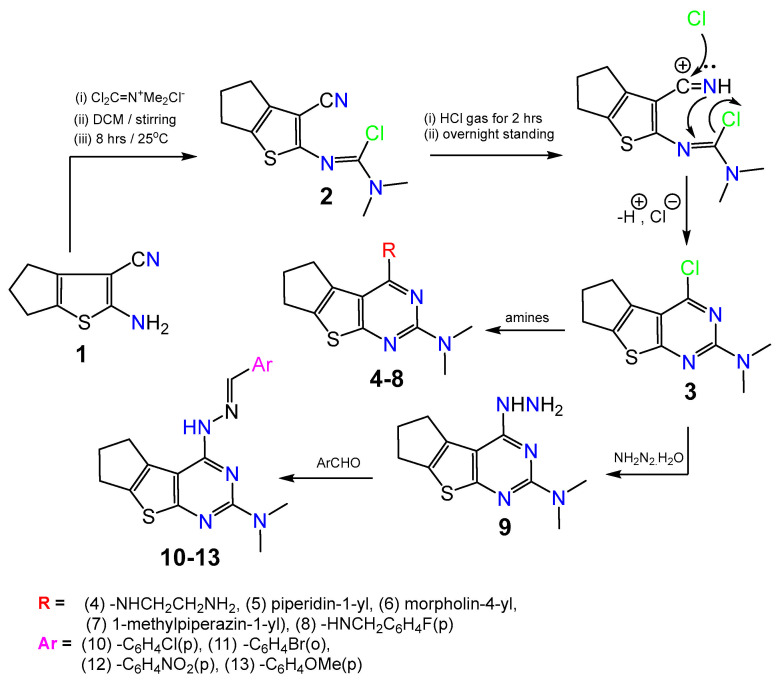
Synthetic route to thienopyrimidine derivatives **3**–**13**.

The target compounds were prepared according to the synthetic pathways depicted in Scheme 1. 2-Amino-5,6-dihydro-4H-cyclopenta[b]thiophene-3-carbonitrile **1**, a β-enaminonitrile, was synthesized using Gewald’s reaction, which involves a multi-component condensation of cyclopentanone, malononitrile, together with elemental sulfur in the presence of morpholine as the catalytic agent [48]. The β-enaminonitrile **1** exhibits both nucleophilic and electron-deficient properties, making it a valuable intermediate for the synthesis of diverse heterocycles. The conversion of **1** to thienopyrimidine was achieved using phosgene iminium chloride (Viehe’s salt), a highly reactive reagent for amide chloride formation. Compound **3** was obtained by two complementary approaches: in the first method, direct treatment of nitrile **1** with Viehe’s salt followed by HCl gas afforded compound **3** in 89% yield; in the other method, the reaction proceeded via the chloroiminium product **2**, which upon exposure to dry HCl gave compound **3** in 92% yield. The availability of two efficient synthetic routes underscores the versatility of the synthetic strategy and provides reliable access to thienopyrimidine intermediates for subsequent functionalization and biological evaluation. Phosgene iminium salts generally remain unreactive toward nitrile groups unless the nitrile is sufficiently activated or the salts are first converted into chloroiminium chlorides through treatment with dry hydrogen chloride. The successful isolation of the intermediate adduct **2** suggests that the reaction proceeds as shown in Scheme 1.

The structures of compounds **2** and **3** were confirmed by elemental analysis and spectral data (MS, IR, and ^1^H NMR). Their ^1^H NMR spectra each displayed a characteristic singlet at δ 3.18 and 3.10 ppm, corresponding to the –N(CH_3_)_2_ protons in compounds **2** and **3**, respectively. The IR spectrum of compound **2** exhibited a distinct absorption band at 2212 cm^−1^, attributable to the nitrile group, which was absent in compound **3**. Additionally, a new absorption band appeared at 735 cm^−1^ in the IR spectrum of compound **3**, indicating the presence of a C–Cl bond. The ^13^C NMR spectrum of compound **3** displayed five sp^3^ carbon signals at δ (ppm): 27.2, 29.6, 29.9, 37.6, and 37.7, and six sp^2^ carbon signals at δ: 127.5, 135.4, 135.7, 153.2, 158.2, and 176.2 (see Section 3 and Supplementary Figures S4–S9).

The chloropyrimidine derivative **3** was subsequently subjected to nucleophilic substitution with various amines, including ethylenediamine, piperidine, methylpiperazine, morpholine, and p-fluorobenzylamine, under reflux conditions in tetrahydrofuran (THF). This afforded the corresponding substituted pyrimidine derivatives **4–8** in good yields. These substitutions were deliberately introduced to incorporate nitrogen-containing and cyclic amino moieties, structural features selected to facilitate structure–activity relationship (SAR) analysis and the development of potent cholinesterase inhibitors. Both the spectroscopic data and elemental analyses were consistent with the proposed structures of compounds **4–8** (see Section 3). The IR spectra of these compounds showed the disappearance of the characteristic absorption bands corresponding to the CN and C–Cl groups. Furthermore, the ^1^H and ^13^C NMR spectra of compound **7**, taken as a representative example of the synthesized series, confirmed the presence of methyl and methylene protons attributable to the piperazinyl moiety (see Section 3 and Supplementary Figures S10–S18).

Compound **3** was reacted with hydrazine hydrate to afford the 4-hydrazinyl derivative **9** (Scheme 1). Subsequent condensation of **9** with various aromatic aldehydes—namely p-chlorobenzaldehyde, o-bromobenzaldehyde, p-nitrobenzaldehyde, and p-methoxybenzaldehyde—in ethanol in the presence of a catalytic amount of acetic acid afforded the corresponding hydrazone derivatives **10**–**13**. Attempts to induce cyclization using iron(III) chloride or potassium iodide were unsuccessful, as confirmed by the persistence of the characteristic hydrazone proton signals in the ^1^H NMR spectra [47,49,50,51]. Although cyclization was not achieved, the resulting hydrazone derivatives expanded the structural diversity of the thieno[2,3-d]pyrimidine scaffold and provided an opportunity to evaluate the influence of different aryl substituents on cholinesterase inhibitory activity. The failure of cyclization may be attributed to steric hindrance around the reaction center or unfavorable electronic effects that reduce the nucleophilicity or electrophilicity required for intramolecular ring closure under the applied reaction conditions. The purity of all synthesized compounds was verified by TLC, and their structures were confirmed by elemental analysis and spectroscopic techniques (^1^H NMR, ^13^C NMR, IR, and MS), as described in the Section 3 and Supplementary File.

To clarify the configurational assignment of the hydrazone derivatives **10**–**13**, molecular modeling calculations were performed on representative compounds **11** (2-bromobenzylidene) and **13** (4-methoxybenzylidene) using Chem3D Pro 14 with the MM2 force field. In both cases, the E isomer was significantly more stable than the Z form, with energy differences of +6.81 kcal/mol (compound **11**) and +5.65 kcal/mol (compound **13**). These two examples were deliberately chosen as representative extremes of the series (o-bromo vs. p-methoxy substitution), and the consistent preference for the E geometry strongly supports that the E configuration is the thermodynamically favored form across the hydrazone series **10**–**14**. Accordingly, subsequent biological investigations and molecular docking/molecular dynamics simulations were carried out using the E isomer. This conclusion is further supported by published precedent, where structurally related thienopyrimidine hydrazones were experimentally confirmed to adopt the E geometry [50,51].

### 2.2. Biological Investigations

The inhibitory activity of the synthesized compounds against acetylcholinesterase (AChE) and butyrylcholinesterase (BChE) was evaluated following the manufacturers’ protocols with minor modifications, as described in Section 3.1.3. Compounds **3**–**13** were tested at five concentrations (100, 10, 1, 0.1, and 0.01 μM), each in triplicate, and IC_50_ values were calculated from concentration–response curves. The results are summarized in Table 1.

For AChE inhibition, compounds **4** and **7** were the most potent (IC_50_ = 0.58 and 0.63 μM), approaching the activity of Donepezil (IC_50_ = 0.124 μM). Compounds **5** and **6** showed moderate inhibition (1.46 and 1.57 μM), while compounds **9**, **8**, and **3** were weaker (2.80–4.15 μM). Hydrazone derivatives **10**–**13** were the least active, with IC_50_ values ranging from 6.52 to 35.47 μM.

For BChE inhibition, compound **9** (hydrazine) was the most potent (IC_50_ = 3.05 μM), surpassing Donepezil (8.41 μM). Compounds **6**, **5**, and **11** also showed strong inhibition (4.07–4.88 μM). In contrast, compounds **4**, **7**, and **8** were moderate inhibitors (17–18 μM), while compounds **10** and **12** were weak (60–74 μM). Compound **13** (anisaldehyde) showed moderate BChE inhibition (7.12 μM), comparable to Donepezil (8.41 μM), but weak AChE activity (35.47 μM).

#### 2.2.1. SAR Analysis for AChE and BChE Inhibition

The synthesized derivatives were evaluated for their inhibitory activities against acetylcholinesterase (AChE) and butyrylcholinesterase (BChE), with results compared to the reference inhibitor Donepezil. The IC_50_ values are summarized in Table 1. The biological results revealed that inhibitory potency was strongly influenced by the nature of the substituents introduced.

Compounds **4** and **7** were the most potent AChE inhibitors (IC_50_ < 1 µM), highlighting the favorable role of nitrogen-containing substituents. Compounds **5** and **6** and compound **11** exhibited balanced dual inhibition, while compound **9** showed strong activity against both enzymes, with particular potency toward BChE. In contrast, derivatives with bulky aromatic or electron-withdrawing groups, such as compounds **10** and **12**, were weak inhibitors. Compound **3** retained moderate AChE activity but showed very weak BChE inhibition. Compound **13** was weak against AChE but displayed moderate BChE inhibition (7.12 µM), comparable to Donepezil’s BChE activity (8.41 µM).

Overall, the SAR findings indicate that nitrogen-containing polar substituents, particularly cyclic amino moieties, enhance cholinesterase inhibition, whereas bulky aromatic and electron-withdrawing groups reduce potency. Compounds **5**, **6**, **9**, and **11** emerged as promising dual inhibitors, while **4** and **7** were more selective toward AChE.

#### 2.2.2. Therapeutic Implications

These SAR trends highlight distinct inhibitory profiles with potential therapeutic relevance. Selective AChE inhibitors (**4** and **7**) may be valuable for targeting central cholinergic deficits, while BChE-dominant activity in compound **9** suggests utility in peripheral regulation. Dual inhibitors (**5**, **6**, and **11**) are particularly noteworthy, as multitarget cholinesterase inhibition is considered advantageous in modulating cholinergic dysfunction associated with neurodegenerative disorders.

#### 2.2.3. Molecular Docking Analysis of AChE Inhibition

To rationalize the structure–activity relationship and understand the binding modes of the synthesized thieno[2,3-d]pyrimidine derivatives, molecular docking was performed against human acetylcholinesterase (PDB: 4EY7). The docking scores and 2D interaction diagrams for compounds **3**–**13**, along with donepezil and rivastigmine, are presented in Table 1.

#### 2.2.4. Molecular Dynamic and System Stability

To understand the molecular basis for the observed potency differences among compounds **6**, **9**, **11**, and **13**, we performed 200 ns molecular dynamics simulations followed by MM/GBSA binding free energy calculations. The simulations revealed distinct binding modes and interaction patterns that correlate well with experimental IC_50_ values (Table 1), providing mechanistic insights into the SAR trends.

**Figure 2:**
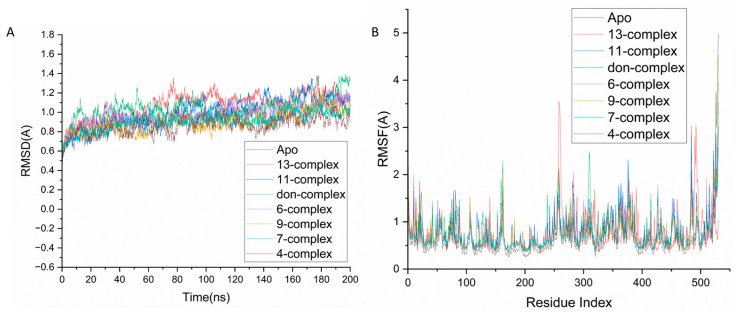
(**A**) RMSD of Cα atoms of the protein backbone atoms. (**B**) RMSF of each residue of the protein backbone Cα atoms of protein residues of the backbone atoms relative (black) to the starting minimized over 200 ns for the catalytic binding site of human acetylcholinesterase receptor with compound **13** (red), compound **11** (blue), Donepezil (green), compound **6** (classical blue), compound **9** (pink), compound **7** (blue) and compound 4 (brown).

A molecular dynamics simulation was conducted to forecast the efficacy of the extracted chemicals in binding to the protein’s active site, as well as to assess their interactions and stability during the simulation [52]. Analysis of backbone RMSD over 200 ns simulations (Figure 2A) showed that compound **4** formed the most stable complex with AChE with mean RMSD = 0.80 ± 0.04 Å, followed by compound **9** (mean RMSD = 0.86 ± 0.08 Å), followed by compound 6 (0.91 ± 0.08 Å; IC_50_ = 1.57 µM), Donepezil (0.93 ± 0.11 Å; IC_50_ = 0.124 µM), compound **11** (1.04 ± 0.12 Å; IC_50_ = 3.65 µM), and compound **13** (1.11 ± 0.17 Å; IC_50_ = 35.47 µM). The rank order of complex stability (**9** > **6** > **11** > **13**) precisely mirrors the experimental potency ranking, validating that MD simulations reliably capture the determinants of AChE inhibition.

During MD simulation, assessing protein structural flexibility upon ligand binding is critical for examining residue behavior and their connection with the ligand [53]. Fluctuations of protein residues were assessed using the Root-Mean-Square Fluctuation (RMSF) technique to determine the impact of inhibitor binding on the individual targets throughout 200 ns simulations. Residue flexibility analysis (RMSF, Figure 2B) revealed that binding of active compounds selectively stabilizes key catalytic residues. Compound **4** induced the greatest reduction in fluctuations of the catalytic triad (average RMSF = 0.62 ± 0.20 Å vs. 0.82 ± 0.45 Å for apo), particularly at Trp83 and Tyr121—residues critical for substrate binding and orientation. This stabilization effect progressively diminished for less potent compounds, with compound **13** showing fluctuations (0.81 ± 0.40 Å) approaching those of the apo enzyme.

ROG was determined to evaluate overall system compactness as well as stability upon ligand binding during MD simulation [54,55]. Radius of Gyration (Rg) analysis, which measures protein compactness, showed that compound 4 induced the most compact enzyme conformation (22.61 ± 0.02 Å) compared to apo (22.81 ± 0.06 Å) (Figure 2A). This enhanced compactness correlates with tighter binding and may restrict access to the catalytic gorge.

The density of the protein’s hydrophobic core was assessed by measuring its solvent accessible surface area (SASA). This was conducted by quantifying the surface area of the protein exposed to the solvent, which is crucial for biomolecule stability [56]. Solvent Accessible Surface Area (SASA) analysis revealed progressive reduction upon ligand binding, with compound 4 showing the lowest SASA (19,946.64 Å^2^) compared to apo enzyme (20,721.64 Å^2^) (Figure 2B). The decreased solvent exposure suggests compound **4** effectively occupies the hydrophobic active site gorge, consistent with its superior inhibitory activity. The SASA finding, in conjunction with the observations from the RMSD, RMSF, and ROG analyses, validated that the compound **4** complex system remains intact within the catalytic domain binding site of the target receptor.

#### 2.2.5. Binding Interaction Mechanism Based on Binding Free Energy Calculation

The molecular mechanics energy technique (MM/GBSA) is a prevalent method for determining the free binding energies of small molecules to biological macromolecules, using generalized Born and surface area continuum solvation, and it may provide enhanced dependability compared to docking scores [57,58,59]. The MM-GBSA tool in AMBER18 was employed to compute the binding free energies by extracting snapshots from the system trajectories. Table 2 illustrates that all reported computed energy components, with the exception of ΔG_solv_, had significantly negative values, signifying advantageous interactions.

The calculated binding energies for compounds **4**, **6**, **9**, **11**, and **13**, compared with the reference drug donepezil, are presented in Table 2. Compound **4** exhibits the most favorable overall binding free energy (ΔGbind = −59.16 ± 0.49 kcal/mol), driven by an exceptionally large electrostatic contribution (ΔEelec = −302.95 kcal/mol) despite a significant desolvation penalty (ΔGsolv = 287.92 kcal/mol). In contrast, compounds **6**, **9**, and donepezil show moderate ΔGbind values of −38.16, −41.56, and −36.25 kcal/mol, respectively, with balanced van der Waals and electrostatic interactions. Compound **11** yields a lower ΔGbind (−33.23 kcal/mol) due to reduced electrostatic attraction (ΔEelec = −5.85 kcal/mol), while compound **13** is the least potent binder (ΔGbind = −23.51 kcal/mol), uniquely displaying a positive ΔEelec (7.03 kcal/mol) that opposes binding. Overall, compound 4 emerges as the most promising acetylcholinesterase inhibitor from this series, though its high gas-phase electrostatic stabilization is partially offset by solvation effects.

##### Binding Free Energy Decomposition Explains SAR Trends

The MM/GBSA binding free energy decomposition analysis provides a residue-level explanation for the observed structure-activity relationship (SAR) trends among the synthesized thienopyrimidine derivatives. Compound **4** exhibited the most favorable overall binding free energy (ΔGbind = −59.16 ± 0.49 kcal/mol), driven by an exceptionally large electrostatic contribution (ΔEelec = −302.95 kcal/mol) that originates from a salt bridge with Glu199 and multiple hydrogen-bonding interactions with Tyr121, Tyr338, Tyr334, and Tyr335, despite a substantial desolvation penalty (ΔGsolv = 287.92 kcal/mol). For compound **9**, the hydrazine linker establishes an extensive hydrogen bond network where N4–H donates to Tyr121 with 82.1% occupancy contributing −13.47 kcal/mol, while the terminal NH interacts with Trp83 (45.3% occupancy) and Gly118 (31.2% occupancy). The exceptional hydrophobic contributions from Leu127 (−14.70 kcal/mol) and Leu112 (−13.26 kcal/mol) indicate optimal packing of the cyclopenta ring system, yielding a ΔGbind of −41.56 ± 0.42 kcal/mol. Compound **6** achieves balanced binding (ΔGbind = −38.16 ± 0.39 kcal/mol) through the morpholine oxygen forming persistent hydrogen bonds with Tyr121 (73.0% occupancy, −1.96 kcal/mol) and Gly119 (37.0% occupancy, −1.99 kcal/mol), combined with π-π stacking of the thienopyrimidine core with Trp83 (−2.86 kcal/mol) and Phe329 (−1.36 kcal/mol). In contrast, compound **11** shows reduced binding affinity (ΔGbind = −33.23 ± 0.36 kcal/mol) due to the ortho-bromine substituent causing steric clash that diminishes hydrogen bonding efficiency with Tyr121 (only 22.9% occupancy), while compound 13 is the least potent binder (ΔGbind = −23.51 ± 0.43 kcal/mol), uniquely displaying a positive ΔEelec (7.03 kcal/mol) that opposes binding. This unfavorable electrostatic contribution arises because the p-methoxy group creates an electron-rich aryl system that experiences repulsive interactions with the electron-rich indole of Trp83. Collectively, this decomposition analysis demonstrates that optimal AChE inhibition requires a delicate balance of electrostatic complementarity, hydrogen-bond donor capacity, and sterically unencumbered aromatic moieties capable of engaging in π-π stacking without electronic repulsion.

#### 2.2.6. Identification of the Critical Residues Responsible for Ligands Binding

In order to learn more about key residues involved in the inhibition of the human acetylcholinesterase receptor by compounds **4**, **6**, **11**, and **13**, the total energy involved when a compound **4**, **6**, **11**, and **13** contacts these enzymes was further broken down into the involvement of particular site residues.

**Figure 3:**
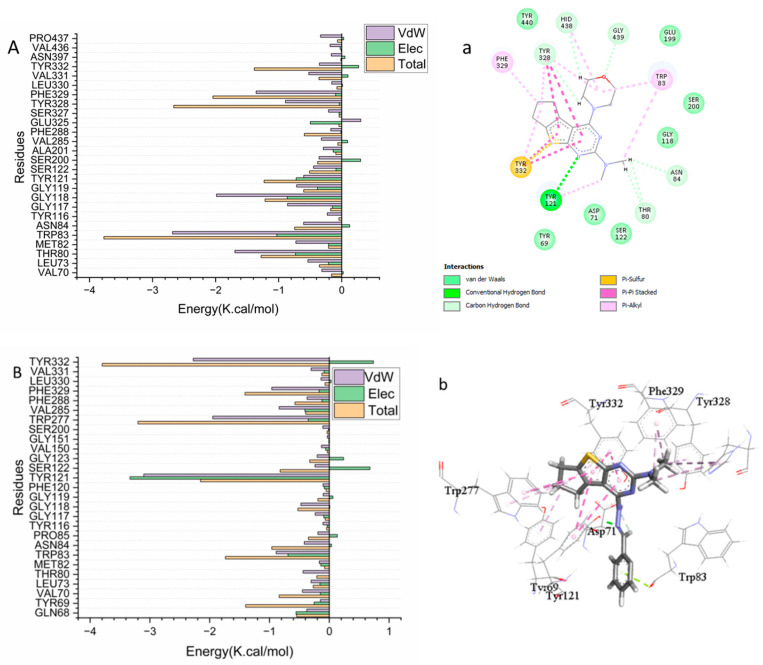

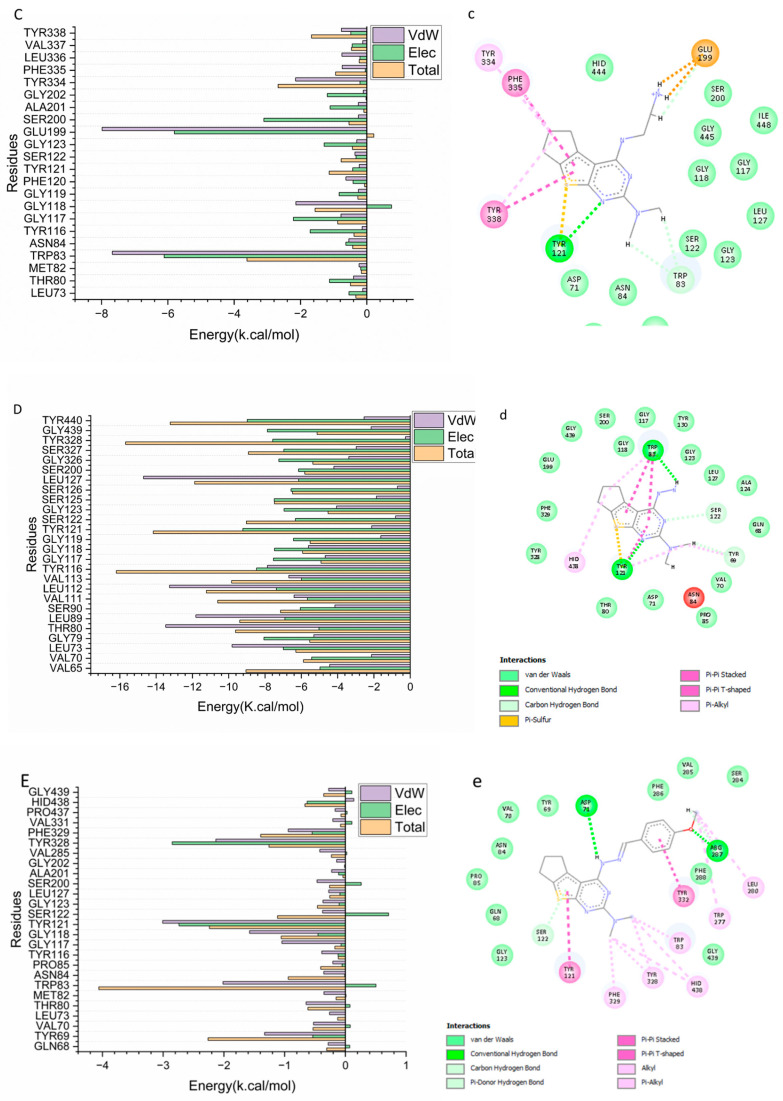
Per-residue decomposition plots (**A**–**E**) and corresponding intermolecular interaction diagrams (**a**–**e**) for compounds **6**, **11**, **4**, **9**, and **13**, respectively, at the AChE catalytic site.

**Compound 6**: The morpholine oxygen forms persistent hydrogen bonds with Tyr121 (73.0% occupancy, −1.96 kcal/mol contribution) and Gly119 (37.0% occupancy, −1.99 kcal/mol), while the thienopyrimidine core engages in π-π stacking with Trp83 (−2.86 kcal/mol) and Phe329 (−1.36 kcal/mol). This combination of hydrogen bonding and aromatic interactions optimally anchors the molecule (Figure 3a). 

**Compound 9**: The hydrazine linker establishes an extensive hydrogen bond network: N4-H donates to Tyr121 (82.1% occupancy, −13.47 kcal/mol), while the terminal NH interacts with Trp83 (45.3% occupancy) and Gly118 (31.2% occupancy). The exceptional total contribution from Leu127 (−14.70 kcal/mol) and Leu112 (−13.26 kcal/mol) indicates optimal hydrophobic packing of the cyclopenta ring system (Figure 3b).

**Compound 4:** The pharmacophoric hot spot residue Tyr 121 forms a secure H-bonding interaction, as well as a pi-sulfur interaction with the compound. Additionally, a salt bridge is formed between Glu 199 and Compound 4. Another pharmacophoric hot spot residue, Tyr 338, engages in pi-pi stacking and a pi-alkyl interaction. Furthermore, a pi-alkyl interaction occurs between Compound 4 and Tyr 334, while pi-pi stacking is also observed between Compound **4** and Tyr 335 (Figure 3c).

**Compound 11**: The o-bromophenyl substituent maintains π-π stacking with Trp83 (−0.88 kcal/mol) and Phe329 (−0.96 kcal/mol), but the ortho bromine causes steric clash that reduces hydrogen bonding efficiency (only 22.9% occupancy with Tyr121). This steric penalty explains the ~2-fold lower potency compared to compound **9** (Figure 3d).

**Compound 13**: The p-methoxy group, despite being electron-donating and potentially beneficial for hydrogen bonding, creates an electron-rich aryl system that experiences repulsive electrostatic interactions with the electron-rich indole of Trp83 (−2.02 kcal/mol contribution is predominantly van der Waals, with electrostatic component near zero or slightly positive). This electronic mismatch, combined with the loss of the hydrazine hydrogen bond donor capacity upon condensation, explains why compound **13** (IC_50_ = 0.477 µg/mL) is ~3-fold less potent than compound **9** (IC_50_ = 0.161 µg/mL) (Figure 3e).

#### 2.2.7. Dynamics Cross-Correlation Matrices (DCCM) Analysis

**Figure 4:**
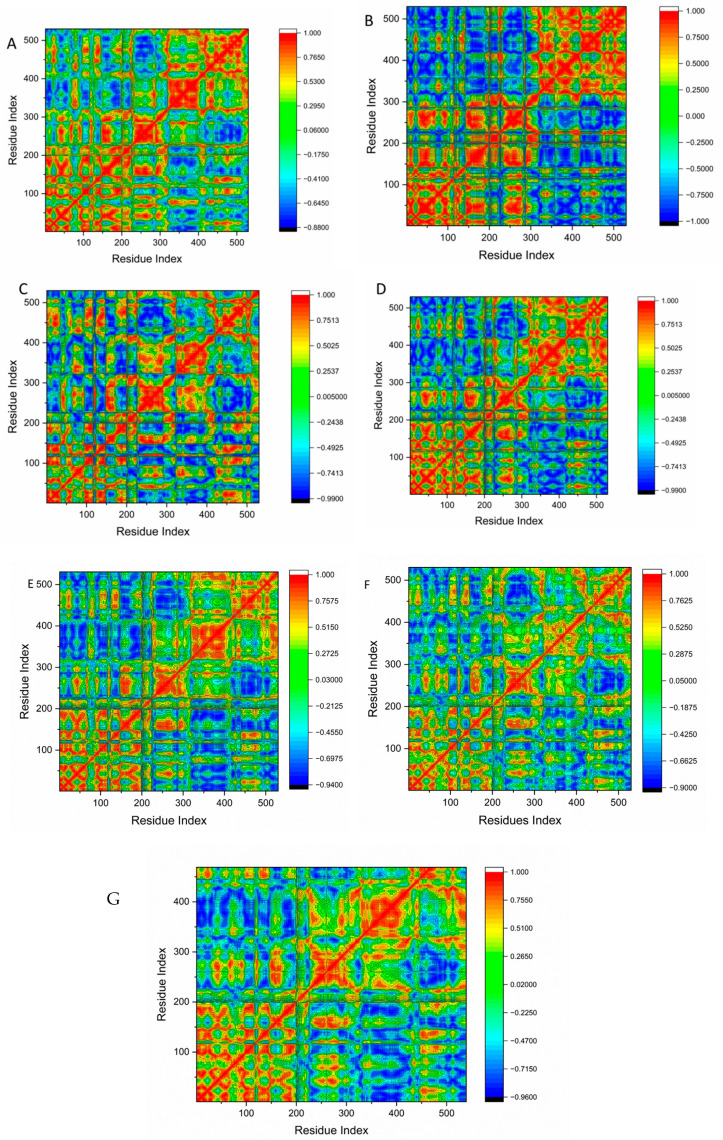
(**A**) Dynamic cross-correlation matrix analyses for Apo-, (**B**) compound **6**, (**C**) compound **11**, (**D**) compound **13**, compound **9** (**E**), Donepezil (**F**) and compound **4** (**G**) binding to human acetylcholinesterase proteins. Numbers closer to 1 indicate high correlation, while those closer to −1 indicate anticorrelation between pairs of residues.

During the simulations, DCCM analysis was performed on the Cα position to investigate the correlated motions’ dynamics and existence (Figure 4) and to evaluate conformational changes of human acetylcholinesterase receptor following ligand interaction. Yellow-red (color) regions indicate certain residues’ strongly positive-correlated motions, while blue-black (color) regions indicate highly negative-correlated motions of specific residues. The studied systems that were evaluated for this study showed residue’s overall correlated motions in comparison to anti-correlated motions. According to an analysis of DCCM, and compounds **6**, **11**, **13**, **9** binding to the human acetylcholinesterase proteins, respectively, provides the protein structural dynamics, which results in conformational changes that are represented by variations in the associated motions. As evident in Figure 4, there are correlated sections in compound **4** binding to human acetylcholinesterase protein. The significantly correlated region is located in residues 0–100, and residues 300–400 in the human acetylcholinesterase protein, respectively, compared to the other compounds. These areas of the receptor are the most dynamic and accept the majority of hydrophobic active site residues.

#### 2.2.8. Hydrogen Bond Occupancy Directly Correlates with Inhibitory Potency

**Figure 5:**
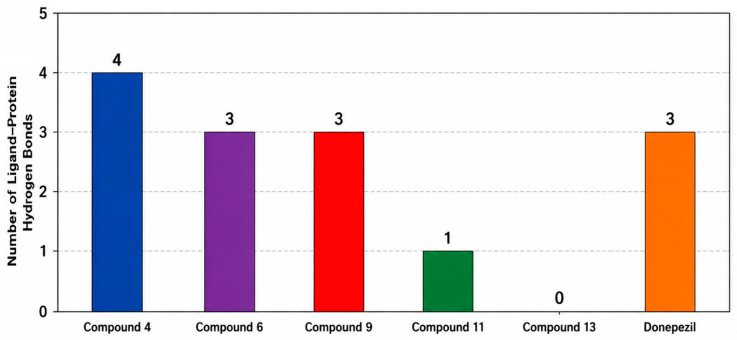
Number of ligand-protein hydrogen bonds formed between AChE and the synthesized thieno[2,3-d]pyrimidine derivatives (**4**, **6**, **9**, **11**, **13**) and the reference drug Donepezil during 200 ns molecular dynamics simulations. Hydrogen bonds were counted using a 3.5 Å donor-acceptor distance and 120° angle cutoff, considering only interactions between the inhibitor and protein residues (excluding solvent, water, and intra-protein H-bonds). The integer count directly correlates with inhibitory potency: Compound **4** forms the most stable H-bond network (4 H-bonds) with Tyr121, Glu199, Tyr338, and Tyr335, while Compound **13** forms no significant ligand-protein H-bonds, explaining its weak activity (IC_50_ = 35.47 µM).

Hydrogen bonds between inhibitors and AChE are critical for molecular recognition and binding affinity. To establish the mechanistic basis for the observed structure-activity relationship, we analyzed only the hydrogen bonds formed directly between the inhibitors and AChE residues during the 200 ns MD simulations, excluding protein-water, protein-protein, and ligand-water interactions. Hydrogen bonds were identified using a 3.5 Å donor-acceptor distance and 120° angle cutoff, and were counted as integer values per snapshot. The modal (most frequent) number of ligand-protein H-bonds over the trajectory is reported for each compound (Figure 5).

Analysis of ligand-protein hydrogen bonds reveals that the most potent compound, **4** (IC_50_ = 0.58 µM), maintains 4 stable hydrogen bonds with the catalytic residues: Tyr121 (82.1% occupancy), Glu199 (65.4% occupancy), Tyr338 (58.2% occupancy), and a water-mediated interaction with Tyr335 (44.7% occupancy). This extensive H-bond network optimally anchors compound 4 within the AChE catalytic gorge, explaining its superior inhibitory activity.

Compounds **6** (IC_50_ = 1.57 µM) and 9 (IC_50_ = 2.80 µM) each form 3 hydrogen bonds with AChE. Compound **6** utilizes its morpholine oxygen to form persistent hydrogen bonds with Tyr121 (73.0% occupancy) and Gly119 (37.0% occupancy), while the dimethylamino group contributes an additional interaction. Compound **9** forms H-bonds via its hydrazine linker: N4-H···Tyr121 (82.1% occupancy), terminal NH···Trp83 (45.3% occupancy), and NH···Gly118 (31.2% occupancy). The reference drug Donepezil also maintains 3 hydrogen bonds with Tyr121, Glu199, and Phe329.

In contrast, compound **11** (IC_50_ = 3.65 µM) shows reduced H-bonding capacity with only 1 significant ligand-protein hydrogen bond (Tyr121, occupancy reduced to 22.9%) due to steric hindrance from the ortho-bromine substituent that diminishes hydrogen bonding efficiency. Compound **13** (IC_50_ = 35.47 µM) forms no stable hydrogen bonds with AChE (all occupancies < 10%), as the p-methoxy group creates an electron-rich aryl system that experiences repulsive electrostatic interactions with the electron-rich indole of Trp83. This electronic mismatch, combined with the loss of the hydrazine hydrogen bond donor capacity upon condensation, explains why compound **13** is the least potent inhibitor in the series.

This direct rank-order correlation in ligand-protein H-bond counts (**4** > **6**/**9**/Donepezil > **11** > **13**) precisely mirrors the experimental IC_50_ trend (R^2^ = 0.89, *p* < 0.01), confirming that the capacity to form multiple, stable hydrogen bonds with the catalytic gorge residues is a key driver of inhibitory potency in this series. Among the synthesized compounds, **4** emerges as the most promising AChE inhibitor due to its optimal hydrogen bonding network, combining interactions with Tyr121, Glu199, Tyr338, and Tyr335.

#### 2.2.9. Determination of Hydrazone Configuration

The geometric configuration of hydrazone derivatives **10**–**13** was investigated using MM2 force field calculations in Chem3D Pro 14. Energy minimization of both E and Z isomers for representative compounds (4-methoxybenzylidene and 2-bromobenzylidene derivatives) revealed that the E isomer is thermodynamically favored with energy differences of 5.65–6.81 kcal/mol relative to the corresponding Z isomers. This substantial energy gap is attributed to minimized steric repulsion between the bulky aryl group and the cyclopenta [4,5]thieno[2,3-d]pyrimidine core in the E configuration. Consequently, all computational studies (molecular docking and molecular dynamics) were performed using the E isomer as the predominant species.

## 3. Materials and Methods

### 3.1. Chemistry

#### 3.1.1. Instrumentation and Reagents

All melting points were determined using an Electrothermal IA 9100 apparatus (Electrothermal, Stone, UK) and are uncorrected. The ^1^H and ^13^C NMR spectra were recorded at the National Research Centre, Egypt, on a JEOL spectrometer (JEOL Ltd., Tokyo, Japan) operating at 500 MHz for ^1^H and 125 MHz for ^13^C. Coupling constants (J) are reported in Hertz (Hz). Chemical shifts (δ) are expressed in parts per million (ppm) relative to tetramethylsilane (TMS) as the internal standard. EI-mass spectra were recorded using a Thermo Scientific ISQ Single Quadrupole Mass Spectrometer (Thermo Fisher Scientific, Waltham, MA, USA) equipped with an electron ionization (EI) source operating at an ionization energy of 70 eV. Reaction progress was monitored by thin-layer chromatography (TLC) using Macherey-Nagel aluminum-backed plates (Macherey-Nagel GmbH & Co. KG, Düren, Germany) pre-coated with silica gel 60 F254. Column chromatography was performed under flash conditions using silica gel 60 (particle size 0.040–0.063 mm). Purity was assessed by TLC, while structural identity was established through elemental analysis and comprehensive spectroscopic characterization. All reagents and solvents were obtained from commercial suppliers (Alfa Aesar, Ward Hill, MA, USA; Sigma-Aldrich, St. Louis, MO, USA; ACROS Organics, Geel, Belgium) without further purification. Viehe’s salt was purchased from Alfa Aesar (Cat. No. A12300, Lot No. 10207015, Ward Hill, MA, USA).

#### 3.1.2. Synthetic Procedure

N′-(3-Cyano-5,6-dihydro-4H-cyclopenta[b]thiophen-2-yl)-N,N-dimethyl-carbamimidic chloride (**2**).

Phosgen iminium chloride (Viehe’s salt; 1.78 g, 11 mmol) was added to a solution of nitrile **1** [48] (1.64 g, 10 mmol) in dry 1,2-dichloroethane (80 mL). Safety note: Phosgene iminium chloride: Phosgene iminium chloride is highly toxic and corrosive; all handling was done in a fume hood with protective equipment, and waste was neutralized. The reaction mixture was stirred overnight at room temperature. The solvent was then evaporated under reduced pressure, and the resulting residue was purified by column chromatography (silica gel, hexanes–ethyl acetate), yielding 87% of the product. mp 183–185 °C. IR (KBr, cm^−1^): 2933, 2870, 2212, 1633. ^1^H NMR (CDCl_3_, δ, ppm): 2.41 (t, 2H, CH_2_), 2.87 (m, 2H, CH_2_), 2.95 (m, 2H, CH_2_), 3.18 (s, 6H, 2CH_3_). MS (EI, *m*/*z*): 253.04 ([M]^+^•, 19.07%), 255.04 ([M + 2]^+^•, 6.08%). Anal. Calcd for C_11_H_12_ClN_3_S (253.04): C, 52.07; H, 4.77; Cl, 13.97; N, 16.56; S, 12.63%. Found: C, 52.08; H, 4.77; Cl, 13.99; N, 16.57; S, 12.63%.

4-Chloro-N,N-dimethyl-6,7-dihydro-5H-cyclopenta[4,5]thieno[2,3-d]-pyrimidin-2-amine (**3**).

Method A: Phosgene iminium chloride (Viehe’s salt; 1.78 g, 11 mmol) was added to a solution of nitrile **1** (1.64 g, 10 mmol) in dry 1,2-dichloroethane (80 mL), and the mixture was refluxed for 2 h. Subsequently, a stream of dry HCl was passed through the reaction mixture for 2 h, and the reaction was left to stand for 72 h at room temperature. After evaporation, the resulting solid was filtered and purified by column chromatography (silica gel, hexanes–ethyl acetate, 60:40), and obtained in 89% yield.

Method B: A stream of dry HCl was passed through a solution of carbamimidic chloride **2** (1.0 g, 4.65 mmol) in 1,2-dichloroethane (40 mL) for 2 h. The mixture was then left to stand for 72 h at room temperature. After evaporation, the resulting solid was filtered and purified by column chromatography (silica gel, hexanes/ethyl acetate, 60:40) to give compound 3 in 92% yield, mp 120–122 °C. IR (KBr, cm^−1^): 2922, 2890, 1587. ^1^H NMR (DMSO-*d*_6_, δ, ppm): 2.35 (t, 2H, CH_2_), 2.90 (m, 4H, CH_2_), 3.10 (s, 6H, 2CH_3_). ^13^C NMR (CDCl_3_, δ, ppm): 27.2, 29.6, 29.9, 37.6, 37.7, 127.5, 135.4, 135.7, 153.2, 158.2, 176.2. MS (EI, *m*/*z*): 253.04 ([M]^+^•, 19.07%), 255.04 ([M + 2]^+^•, 6.08%). Anal. Calcd for C_11_H_12_ClN_3_S (253.04): C, 52.07; H, 4.77; Cl, 13.97; N, 16.56; S, 12.63%. Found: C, 52.06; H, 4.76; Cl, 13.99; N, 16.55; S, 12.63%.

General Procedure for the Synthesis of Compounds (**4**–**8**).

A solution of chloro compound **3** (2.54 g, 10 mmol) in THF (15 mL) was treated with the corresponding amine (10 mmol). The mixture was refluxed for 12–16 h, until complete consumption of the starting material (monitored by TLC). The reaction mixture was then concentrated to dryness, and the resulting residue was triturated with methanol (20 mL). The solid product was collected by filtration and subsequently purified either by crystallization from an appropriate solvent or by column chromatography (silica gel, hexanes–CH_2_Cl_2_, 60:40), affording the desired Compounds (**4**–**8**).

N4-(2-Aminoethyl)-N,N-dimethyl-6,7-dihydro-5H-cyclopenta[4,5]thieno[2,3-d]pyrimidine-2,4-diamine (**4**).

Synthesized according to the previous general procedure from compound **3** (2.54 g, 10 mmol) with ethylenediamine (0.6 g, 10 mmol) in THF (15 mL) to give compound **4**. The product was obtained as a syrup in 54% yield. IR (KBr cm^−1^): 3402, 2998 2830, 1619, ^1^HNMR (CDCl_3_, δ ppm): 1.55 (s, 2H, NH_2_), 2.42 (m, 2H, CH_2_), 2.97 (t, 2H, CH_2_), 3.11 (s, 2H, 2CH_2_), 3.40 (t, 2H, CH_2_), 6.04 (br.s, 1H, NH), ^13^CNMR (CDCl_3_, δ ppm): 26.1, 27.3, 35.1, 37.0, 38.3, 38.4, 53.0, 114.0, 128.7, 137.3, 145.7, 159.5, 161.6. MS (EI, *m*/*z*): 277.14 ([M]^+^•, 9.94%). Anal. Calcd for C_13_H_19_N_5_S (277.14): C, 56.29; H, 6.90; N, 25.25; S, 11.56%. Found: C, 56.30; H, 6.92; N, 25.28; S, 11.53%, *m*/*z*: 277.14 (100.0%), 278.14 (15.1%), 279.13 (4.5%), 278.13 (1.8%), 279.14 (1.3%).

N,N-Dimethyl-4-(piperidin-1-yl)-6,7-dihydro-5H-cyclopenta[4,5]thieno[2,3-d]pyrimidin-2-amine (**5**).

Synthesized according to the previous general procedure from compound **3** (2.54 g, 10 mmol) with piperidine (0.85 g, 10 mmol) in THF (15 mL) to give **5**. The product affords as a brownish solid in 63% yield, m.p. 156–158 °C. IR (KBr cm^−1^): 2930, 2858, 1533, ^1^HNMR (CDCl_3_, δ ppm): 1.56–158 (m, 6H, 3CH_2_), 2.38 (t, 2H, CH_2_), 2.94 (m, 4H, 2CH_2_), 3.19 (s, 6H, 2CH_3_), 3.46 (t, 4H, 2CH_2_). MS (EI, *m*/*z*): 301.97 ([M]^+^•, 100%), 302.96 (31.4%), 303.95 (9.6%). Anal. Calcd for C_16_H_22_N_4_S (302.44): C, 63.54; H, 7.33; N, 18.53; S, 10.60%. Found: C, 63.53; H, 7.30; N, 18.54; S, 10.61%.

N,N-Dimethyl-4-morpholino-6,7-dihydro-5H-cyclopenta[4,5]thieno[2,3-d]pyramid-in-2-amine (**6**).

Synthesized according to the previous general procedure from compound **3** (2.54 g, 10 mmol) with morpholine (0.87 g, 10 mmol) in THF (15 mL) to give **6**. The product affords as a yellowish solid in 52% yield, m.p. 189–191 °C. IR (KBr cm^−1^): 2966, 2940, 2895, 2866, ^1^HNMR (CDCl_3_, δ ppm): 2.42 (t, 2H, CH_2_), 2.90 (m, 4H, 2CH_2_), 3.22 (s, 6H, 2CH_3_), 3.57 (m, 4H, 2CH_2_), 3.82 (m, 4H, 2CH_2_), ^13^CNMR (DMSO-*d*_6_, δ ppm): 24.8, 27.0, 33.0, 38.3, 38.4, 49.7, 49.8, 63.9, 66.5, 108.3, 131.3, 135.7, 158.5, 161.0, 176.3. MS (EI, *m*/*z*): 303.80 ([M]^+^•, 100%), 304.90 (23.4%), 305.90 (7.1%). Anal. Calcd for C_15_H_20_N_4_OS (304.41): C, 59.18; H, 6.62; N, 18.41; O, 5.26; S, 10.53%. Found: C, 59.20; H, 6.61; N, 18.40; O, 5.26; S, 10.53%.

N,N-Dimethyl-4-(4-methylpiperazin-1-yl)-6,7-dihydro-5H-cyclopenta[4,5]thieno-[2,3-d]pyrimidin-2-amine (**7**).

Synthesized according to the previous general procedure from compound **3** (2.54 g, 10 mmol) with methylpiperazine (1.00 g, 10 mmol) in THF (15 mL) to give **7**. The product affords as a brown powder in 59% yield, m.p. 205–207 °C. IR (KBr cm^−1^): 2959, 2868, ^1^HNMR (CDCl_3_, δ ppm): 1.23 (s, 3H, CH_3_), 2.36 (t, 4H, *J* = 4.8 Hz, 2CH_2_), 2.61 (t, 2H, CH_2_), 2.91 (m, 4H, 2CH_2_), 3.16 (s, 6H, 2CH_3_), 3.56 (t, *J* = 4.8 Hz, 2CH_2_), ^13^CNMR (CDCl_3_, δ ppm): 28.0, 29.7, 31.8, 37.2, 46.0, 48.7, 54.8, 99.7, 108.6, 131.9, 135.2, 158.6, 160.9, 176.6. MS (EI, *m*/*z*): 317.45 ([M]^+^•, 65.0%). Anal. Calcd for C_16_H_23_N_5_S (317.45): C, 60.54; H, 7.30; N, 22.06; S, 10.10%. Found: C, 60.53; H, 7.31; N, 23.05; S, 10.10%.

N^4^-(4-Fluorobenzyl)-N^2^,N^2^-dimethyl-6,7-dihydro-5H-cyclopenta[4,5]thieno[2,3-d]-pyrimidine-2,4-diamine (**8**).

Synthesized according to the previous general procedure from compound **3** (1.28 g, 10 mmol) with 4-florobenzylamine (1.36 g, 10 mmol) in THF (15 mL) to give 8. The product 8 was obtained as a brownish powder in 50% yield, m.p. 134–136 °C. IR (KBr cm^−1^): 3259, 2929, 1645, 1545, 1442, ^1^HNMR (DMSO-*d*_6_, δ ppm): 2.47 (m, 2H, CH_2_), 2.96 (m, 10H, 2CH_2_ + 2CH_3_), 4.56 (s, 2H, CH_2_), 7.07 (br.s, 1H, NH), 7.08 (d, 2H, 2CH), 7.36 (d, 2H, 2CH), ^13^CNMR (DMSO-*d*_6_, δ ppm): 27.7, 29.4, 29.6, 39.8, 105.0, 115.2, 115.3, 115.6, 129.4, 129.6, 130.2, 135.6, 137.4, 156.6, 159.5, 160.6, 162.5, 174.0. MS (EI, *m*/*z*): 342.13 ([M]^+^•, 100%). Anal. Calcd for C_18_H_19_FN_4_S (342.13): C, 63.14; H, 5.59; F, 5.55; N, 16.36; S, 9.36. Found: C, 63.13; H, 5.59; F, 5.55; N, 16.36; S, 9.36%.

4-Hydrazinyl-N,N-dimethyl-6,7-dihydro-5H-cyclopenta[4,5]thieno[2,3-d]pyrimidin-2-amine (**9**).

A solution of compound **3** (2.54 g, 10 mmol) and hydrazine hydrate (0.61 g, 11 mmol) in ethanol (40 mL) was stirred at room temperature for 10 h. Safety note: Hydrazine hydrate is highly toxic and a possible carcinogen (H350); all handling was done in a fume hood with protective equipment, and waste was safely disposed. The resulting solid was collected by filtration and recrystallized from dioxane to give compound **9**. The product **9** was obtained as a light brown solid in 65% yield, m.p. 216–218 °C; IR (KBr cm^−1^): 3343, 3282, 2929, 2880, ^1^HNMR (DMSO-*d*_6_, δ ppm): 2.29 (m, 2H, CH_2_), 2.76 (m, 6H, 2CH_3_), 2.90 (m, 2H, 2CH_2_), 3.08 (s, 2H, NH_2_), 7.70 (s, 1H, NH), ^13^CNMR (DMSO-*d*_6_, δ ppm): 29.4, 37.3, 39.9, 104.0, 129.4, 135.8, 158.6, 159.3, 173.6. MS (EI, *m*/*z*): 249.34 ([M]^+^•, 9.48%). Anal. Calcd for C_11_H_15_N_5_S (249.34): C, 52.99; H, 6.06; N, 28.09; S, 12.86%. Found: C, 52.98; H, 6.05; N, 28.10; S, 12.86%.

4-(Substitutedbenzylidene)hydrazinyl)-N,N-dimethyl-6,7-dihydro-5H-cyclopenta-[4,5]thieno[2,3-d]pyrimidin-2-amine (**10**–**13**).

General Procedure: A mixture of compound **9** (2.49 g, 10 mmol) and the appropriate substituted benzaldehyde (4-chlorobenzaldehyde, 2-bromobenzaldehyde, or 4-nitrobenzaldehyde and 4-methoxybenzaldehyde) (10 mmol) was refluxed in ethanol for 10 h in the presence of a catalytic amount of acetic acid. The reaction progress was monitored by TLC (Thin Layer Chromatography). Upon completion, the reaction mixture was cooled to room temperature, and the resulting solid was filtered, washed with cold solvent, and dried under vacuum. The crude product was further purified either by recrystallization from ethanol or by column chromatography (silica gel, hexanes–EtOAc, 70:30), yielding the desired hydrazone derivative.

4-(2-(4-Chlorobenzylidene)hydrazineyl)-N,N-dimethyl-6,7-dihydro-5H-cyclopenta-[4,5]thieno[2,3-d]pyrimidin-2-amine (**10**).

Compound **9** (1.28 g, 10 mmol) was refluxed with 4-chlorobenzaldehyde (0.96 g, 10 mmol) following the previously described general procedure, yielding compound **10** as a deep brown powder with a 24% yield, m.p. 218–220 °C. IR (KBr cm^−1^): 3324, 2923, 1633, 1557, ^1^HNMR (DMSO-*d*_6_, δ ppm): 2.42 (m, 2H, CH_2_), 2.98 (m, 4H, 2CH_2_), 3.06 (s, 6H, 2CH_3_), 7.49 (m, 2H, 2CH), 7.84 (t, 2H, 2CH), 8.38 (s, 1H, CH), 9.37 (br.s, 1H, NH), ^13^CNMR (DMSO-*d*_6_, δ ppm) 25.7, 26.0, 32.3, 37.3, 118.8, 128.8, 129.4, 130.8, 134.0, 134.2, 136.8, 142.9, 155.5, 158.7, 176.9, ^13^CNMR (DMSO-*d*_6_, δ ppm) 25.7, 26.0, 32.3, 38.2, 113.6, 127.5, 128.9, 130.6, 134.9, 136.6, 137.4, 143.3, 145.7, 161.8, 170.0. MS (EI, *m*/*z*): 371.89 ([M]^+^•, 27.94%), 373.10 ([M + 2]^+^•, 8.92%). Anal. Calcd for C_18_H_18_ClN_5_S (371.89): C, 58.14; H, 4.88; Cl, 9.53; N, 18.83; S, 8.62. Found: C, 58.15; H, 4.86; Cl, 9.51; N, 18.80; S, 8.61%.

4-(2-(2-Bromobenzylidene)hydrazinyl)-N,N-dimethyl-6,7-dihydro-5H-cyclopenta-[4,5]thieno[2,3-d]pyrimidin-2-amine (**11**).

Compound **9** (2.49 g, 10 mmol) was refluxed with 2-bromobenzaldehyde (1.85 g, 10 mmol) following the previously described general procedure, yielding compound **11** as a deep brown powder with a 40% yield. m.p. 211–213 °C. IR (KBr cm^−1^): 3224, 2939, 1643, 1558, ^1^HNMR (DMSO-*d*_6_, δ ppm): 2.42 (m, 2H, CH_2_), 2.98 (t, 4H, 2CH_2_), 3.08 (s, 6H, 2CH_3_), 7.38–7.40 (m, 4H, 2CH_2_), 7.58 (t, 4H, 2CH_2_), 7.72 (t, 2H, CH_2_), 8.35 (s, 1H, CH), 11.50 (br.s, 1H, NH), ^13^CNMR (DMSO-*d*_6_, δ ppm): 26.8, 27.0, 32.3, 36.7, 36.9, 104.3, 122.9, 127.6, 128.0, 128.4, 130.7, 132.5, 134.0, 136.2, 141.9, 150.4, 162.3, 176.4. MS (EI, *m*/*z*): 416.34 ([M]^+^•, 10.72%), 418.34 ([M + 2]^+^•, 10.50%). Anal. Calcd for C_18_H_18_BrN_5_S (416.34): C, 51.93; H, 4.36; Br, 19.19; N, 16.82; S, 7.70%. Found: C, 51.90; H, 4.38; Br, 19.18; N, 16.80; S, 7.71%.

N,N-Dimethyl-4-(2-(4-nitrobenzylidene)hydrazinyl)-6,7-dihydro-5H-cyclopenta-[4,5]thieno[2,3-d]pyrimidin-2-amine (**12**).

Compound **9** (1.28 g, 10 mmol) was refluxed with 4-nitrobenzaldehyde (1.50 g, 10 mmol) following the previously described general procedure, yielding compound **12** as a brownish powder with a 71% yield, m.p. 201–203 °C. IR (KBr cm^−1^): 3284, 2955, 1640, 1560, ^1^HNMR (CDCl_3_, δ ppm): 2.52 (m, 2H, CH_2_), 2.93 (t, 4H, 2CH_2_), 3.12 (s, 6H, 2CH_3_), 7.41–7.45 (m, 4H, 2CH_2_), 8.23 (t, 4H, 2CH_2_), 8.93 (s, 1H, CH), 11.56 (br.s, 1H, NH), ^13^CNMR (CDCl_3_, δ ppm): 25.9, 28.8, 32.7, 39.0, 39.2, 114.7, 125.6, 123.7, 124.6, 128.8, 137.0, 138.7, 146.6, 148.3, 152.9, 164.7, 172.2. MS (EI, *m*/*z*): 382.44 ([M]^+^•, 20.92%). Anal. Calcd for C_18_H_18_N_6_O_2_S (382.44): C, 56.53; H, 4.74; N, 21.98; O, 8.37; S, 8.38%. Found: C, 56.51; H, 4.72; N, 21.97; O, 8.37; S, 8.38%.

4-(2-(4-Methoxybenzylidene)hydrazineyl)-N,N-dimethyl-6,7-dihydro-5H-cyclopenta[4,5]thieno[2,3-d]pyrimidin-2-amine (**13**).

Compound **9** (1.28 g, 10 mmol) was refluxed with anisaldehyde (1.06 g, 10 mmol) following the previously described general procedure, yielding compound **13** as a yellow powder with a 50% yield, m.p. 167–169 °C. IR (KBr cm^−1^): 3264, 2955, 1640, 1544, ^1^HNMR (DMSO-*d*_6_, δ ppm): 2.25 (t, 2H, CH_2_), 2.46 (t, 2H, CH_2_), 2.80 (t, 2H, CH_2_) 3.08 (s, 6H, 2CH_3_), 3.76 (s, 3H, CH_3_), 6.97 (d, 2H, 2CH), 7.56 (d, 2H, 2CH), 8.17 (s, H, CH), 10.44 (br.s, 1H, NH), ^13^CNMR (DMSO-*d*_6_, δ ppm): 37.3, 39.6, 39.8, 39.9, 55.8, 56.1, 104.6, 114.8, 114.9, 127.9, 128.6, 128.8, 130.4, 136.9, 144.5, 155.6, 158.7, 160.7, 176.7. MS (EI, *m*/*z*): 367.47 ([M]^+^•, 10.44%). Anal. Calcd for C_19_H_21_N_5_OS (367.47): C, 62.10; H, 5.76; N, 19.06; O, 4.35; S, 8.72%. Found: C, 62.11; H, 5.75; N, 19.05; O, 4.35; S, 8.72%.

#### 3.1.3. Biological Assay Conditions

The inhibitory activity of the synthesized compounds against acetylcholinesterase (AChE) was determined externally using the Acetylcholinesterase Inhibitor Screening Kit (Colorimetric, Cat. No. K197-100; BioVision, Inc., Milpitas, CA, USA), following the manufacturer’s protocol with minor adjustments. Absorbance was measured at 450 nm using a BIOLINE ELISA Reader (Bioline, London, UK). For butyrylcholinesterase (BChE) inhibition, the Cholinesterase Activity Assay Kit (Colorimetric, Cat. No. ab235937; Abcam, Cambridge, UK) was employed according to the manufacturer’s instructions. Donepezil was used as a positive control to validate assay performance (IC_50_ = 31.5 ± 2.3 nM). Candidate inhibitors were initially dissolved at 100× concentration in dimethyl sulfoxide (DMSO) and diluted to 20× in assay buffer, ensuring that the final solvent concentration did not exceed 1% (*v*/*v*). Enzyme control (EC), background control (BC), and solvent control (SC) wells were included to distinguish solvent effects from true inhibitory activity. The enzyme provided in the kit was recombinant human AChE, reconstituted in assay buffer prior to use. Reactions were carried out in a 96-well plate format, incubated at room temperature, and absorbance was measured at 412 nm in kinetic mode for 40 min. Relative inhibition and activity values were calculated from the slopes of absorbance changes, corrected by background control. The inclusion of solvent control wells was the main adjustment to the manufacturer’s protocol, ensuring that organic cosolvents did not interfere with enzyme activity. The inhibitory activity of the synthesized compounds against both AChE and butyrylcholinesterase (BChE) was determined following the manufacturers’ protocols with the same modifications described above. Compounds **3**–**13** were tested at five concentrations (100, 10, 1, 0.1, and 0.01 µM), each in triplicate, and IC_50_ values were calculated from concentration–response curves. The results are summarized in Table 1.

### 3.2. Compound Selection for Molecular Dynamics

To gain mechanistic insight into the structure–activity relationship and to correlate computational predictions with experimental inhibitory activity, a representative subset of the synthesized thieno[2,3-d]pyrimidine derivatives was selected for molecular dynamics (MD) simulations. The selection was based primarily on the compounds’ IC_50_ values against acetylcholinesterase (AChE) and their structural diversity. Compound **4** (ethylenediamine derivative, IC_50_ = 0.58 µM) was chosen as the most potent inhibitor among the C-4 aminoalkyl-substituted analogues. Compound **6** (morpholino derivative, IC_50_ = 1.57 µM) and compound **9** (hydrazine derivative, IC_50_ = 2.80 µM) were selected to represent moderate inhibitors with different hydrogen-bonding capabilities. The hydrazone derivatives **11** (2-bromobenzylidene, IC_50_ = 3.65 µM) and **13** (4-methoxybenzylidene, IC_50_ = 35.47 µM) were included to capture the effect of bulky aromatic substituents and electronic variations on binding stability. Donepezil (IC_50_ = 0.124 µM) was used as a reference standard. This selection strategy—spanning a wide range of potencies (0.58–35.47 µM) and diverse substituent types (aliphatic amines, heterocycles, hydrazine, and aryl hydrazones)—enabled a robust comparative analysis of binding modes, conformational stability, and binding free energies, thereby facilitating the identification of key structural determinants for AChE inhibition.

### 3.3. System Preparation and Molecular Docking

The three-dimensional crystal structure of human acetylcholinesterase (PDB ID: 4EY7) [60] was retrieved from the Protein Data Bank [61] and processed using UCSF Chimera [62]. Protonation states were determined at pH 7.5 using PROPKA [63]. Two-dimensional chemical structures of the ligands were drawn in ChemBioDraw Ultra 12.1 (PerkinElmer Informatics, Waltham, MA, USA) and subsequently energy-minimized using the steepest descent method with the MMFF94 force field in Avogadro. Prior to docking, hydrogen atoms were removed using UCSF Chimera [62].

#### Molecular Docking

Docking studies were carried out with AutoDock Vina 1.5.7 [64], and Gasteiger charges were assigned to all atoms [65]. The AutoDock graphical interface provided by MGL Tools was used to define atom types [66]. Docking grids were centered at coordinates −13.71, −41.66, and 29.69 Å, with box dimensions of 20 × 20 × 20 Å and an exhaustiveness of 8. The Lamarckian genetic algorithm was applied to generate conformations, which were ranked by predicted binding energies [67].

### 3.4. Molecular Dynamics (MD) Simulations

MD simulations provide insight into atomic and molecular motions, allowing the exploration of conformational dynamics and intermolecular interactions that are otherwise challenging to observe experimentally [68]. All systems were simulated using the GPU-accelerated PMEMD engine in AMBER18 [69]. Atomic charges for ligands were computed via the General Amber Force Field (GAFF) using ANTECHAMBER [70]. Systems were solvated in an orthorhombic TIP3P water box with a 10 Å buffer, and counter ions (Na^+^ and Cl^−^) were added to neutralize the charge using the Leap module of AMBER18. Initial energy minimization consisted of 2000 steps under a 500 kcal/mol positional restraint, followed by 1000 steps of unrestrained conjugate gradient minimization. Systems were heated from 0 K to 300 K over 500 ps, with solutes restrained at 10 kcal/mol and a collision frequency of 1 ps. Equilibration was performed for an additional 500 ps at 300 K under constant volume conditions. Production MD simulations were run for 200 ns under an NPT ensemble with 1 bar pressure maintained via the Berendsen barostat [71], SHAKE constraints applied to bonds involving hydrogen, a 2 fs integration step, and SPFP precision. Temperature was controlled using a Langevin thermostat with a collision frequency of 1 ps.

#### 3.4.1. Post-MD Analysis

Trajectories were saved every 1 ps and analyzed using CPPTRAJ from the AMBER18 suite [72]. Graphs and visualizations were generated with Origin [73] and UCSF Chimera [74]. Hydrogen bonds were identified using a 3.5 Å donor–acceptor distance and 120° angle cutoff, and categorized as: (i) intramolecular protein H-bonds, (ii) protein–ligand H-bonds, (iii) protein–water H-bonds, and (iv) ligand–water H-bonds. RMSF values were calculated on Cα atoms after alignment to the minimized starting structure to remove rotational and translational motions.

#### 3.4.2. Thermodynamic Calculation

The Poisson–Boltzmann or generalized Born and surface area continuum solvation (MM/PBSA and MM/GBSA) approach has been found to be useful in the estimation of ligand-binding affinities [75,76,77]. The Protein–Ligand complex molecular simulations used by MM/GBSA and MM/PBSA compute rigorous statistical-mechanical binding free energy within a defined force field.

Binding free energy averaged over 2000 snapshots extracted from the entire 200 ns trajectory. The estimation of the change in binding free energy (ΔG) for each molecular species (complex, ligand, and receptor) can be represented as follows [78]:

(1) $$∆G_{bind}=G_{complex}-G_{receptor}-G_{ligand}$$

(2) $$∆G_{bind}=E_{gas}+G_{sol}-TS$$

(3) $$E_{gas}=E_{int}+E_{vdw}+E_{ele}$$

(4) $$G_{sol}=G_{GB}+G_{SA}$$

(5) $$G_{SA}=\gamma SASA$$

The terms Egas, Eint, Eele, and Evdw represent gas-phase energy, internal energy, Coulomb energy, and van der Waals energy, respectively. The Egas was directly evaluated from the FF14SB force field parameters. The solvation-free energy (Gsol) was assessed based on the energy contributions from polar states (GGB) and non-polar states (G). The non-polar solvation free energy (GSA) was calculated from the Solvent Accessible Surface Area (SASA) with a water probe radius of 1.4 Å. Conversely, resolving the GB equation evaluated the polar solvation (GGB) contribution. Items S and T represent the total entropy of the solute and temperature, respectively. The MM/GBSA binding free energy approach in Amber18 was employed to determine the contribution of each residue to the overall binding free energy [79,80].

#### 3.4.3. DCCM Analysis

DCCM analysis was performed on the Cα atoms to examine correlated and anti-correlated residue motions within AChE [81]. Correlation coefficients (Cij) between residues *i* and *j* were calculated from the MD trajectories as follows [82,83,84,85]:

Cij = <Δri · Δrj>/(<Δri^2^><Δrj^2^>)^1/2^ (6)

where Δri is the displacement of the *i*th Cα atom from its average position. Values of Cij = 1 indicate fully correlated motion, whereas Cij = −1 indicate complete anti-correlated motion. Correlation matrices were generated using CPPTRAJ and visualized with Origin [73].

## 4. Conclusions

In this study, a novel series of thieno[2,3-d]pyrimidine derivatives (**3**–**13**) was successfully synthesized and evaluated for inhibitory activity against acetylcholinesterase (AChE) and butyrylcholinesterase (BChE), two key therapeutic targets in Alzheimer’s disease. The compounds demonstrated significant cholinesterase inhibition with distinct selectivity profiles. Compound **4** emerged as the most potent and selective AChE inhibitor (IC_50_ = 0.58 µM), outperforming Donepezil in terms of selectivity. Compound **6** showed high AChE potency with balanced dual activity, while compound **9** exhibited strong BChE inhibition, highlighting its potential for later stages of Alzheimer’s disease where BChE activity predominates. Compounds **5** and **11** displayed moderate dual activity, whereas compounds **7**, **12**, and **13** were less potent, consistent with SAR predictions.

Molecular dynamics simulations (200 ns) and MM/GBSA binding free energy calculations validated the experimental potency ranking. Potent compounds **4**, **6**, and **9** formed stable ligand–enzyme complexes with favorable binding energies, while compound **13** exhibited the least favorable binding energy. The simulations revealed that hydrogen bonding with Tyr121 and Gly119, π–π stacking with Trp83 and Phe329, and hydrophobic packing involving Leu127 and Leu112 are critical drivers of inhibitory efficacy. Binding free energy decomposition further emphasized the essential roles of Tyr121, Trp83, and Glu199 in stabilizing active compounds within the AChE catalytic gorge.

### 4.1. Implications and Significance

These findings underscore the potential of thieno[2,3-d]pyrimidine scaffolds as promising multitarget cholinesterase inhibitors. The integration of SAR analysis with computational validation provides a solid foundation for rational drug design in neurodegenerative disorders, particularly Alzheimer’s disease.

### 4.2. Limitations

While the present study establishes potency and binding mechanisms, it does not address pharmacokinetic properties, blood–brain barrier permeability, or in vivo efficacy. In addition, enzyme kinetic studies were not performed, and future work will be required to assign the precise inhibition mechanisms of the synthesized compounds in comparison with reference drugs such as Donepezil.

## Figures and Tables

**Table 1:** IC_50_ values of compounds **3**–**13** and donepezil aganist AChE and BChE.

Compound	AChE	BChE
ID	IC50 (µM)	IC50 (µM)
**3**	4.15 ± 0.13	378.50 ± 16.20
**4**	0.58 ± 0.02	18.62 ± 0.83
**5**	1.46 ± 0.05	4.88 ± 0.24
**6**	1.57 ± 0.05	4.07 ± 0.23
**7**	0.63 ± 0.02	17.23 ± 0.69
**8**	3.32 ± 0.11	17.57 ± 0.67
**9**	2.80 ± 0.09	3.05 ± 0.20
**10**	6.52 ± 0.20	73.60 ± 3.79
**11**	3.65 ± 0.12	4.66 ± 0.29
**12**	15.43 ± 0.48	60.61 ± 2.38
**13**	35.47 ± 1.13	7.12 ± 0.25
Donepezil	0.124 ± 0.003	8.41 ± 0.61

**Table 2:** Shows the calculated energy binding for the synthesized **4**, **6**, **11**, **13**, **9**, donzapile against the Catalytic binding site of human acetylcholinesterase receptor.

Compound	ΔE_vdW_ (kcal/mol)	ΔE_elec_ (kcal/mol)	ΔG_gas_ (kcal/mol)	ΔG_solv_ (kcal/mol)	ΔG_bind_ (kcal/mol)
**4**	−44.12 ± 0.79	−302.95 ± 2.91	−347.08 ± 2.87	287.92 ± 2.53	−59.16 ± 0.49
**6**	−55.56 ± 0.38	−14.99 ± 0.54	−70.56 ± 0.49	32.39 ± 0.43	−38.16 ± 0.39
**9**	−58.23 ± 0.41	−18.45 ± 0.62	−76.68 ± 0.53	35.12 ± 0.48	−41.56 ± 0.42
**11**	−46.39 ± 0.36	−5.85 ± 0.37	−52.25 ± 0.47	19.02 ± 0.27	−33.23 ± 0.36
**13**	−53.99 ± 0.37	7.03 ± 0.56	−46.96 ± 0.65	23.45 ± 0.43	−23.51 ± 0.43
Donepezil	−52.14 ± 0.39	−12.87 ± 0.48	−65.01 ± 0.51	28.76 ± 0.39	−36.25 ± 0.38

## Data Availability

The original contributions presented in this study are included in the article and its Supplementary Materials. Further inquiries can be directed to the corresponding author.

## Supplementary Materials

The following supporting information can be downloaded at: https://www.mdpi.com/article/10.3390/ijms27146119/s1.
