# Supplementary material for: Synthesis, Biological Evaluation, Molecular Docking and Molecular Dynamics of Substituted Thieno[2,3-d]pyrimidine Derivatives as Potential Anti-Alzheimer Agents
Source: Int J Mol Sci. 2026 Jul 8;27(14):6119. doi: 10.3390/ijms27146119 (PMC13411822; doi:10.3390/ijms27146119)
Supplement: Supplementary file 1 [file ijms-27-06119-s001.zip › ijms-4289863-supplementary-final.pdf]

Supporting information

Synthesis, Biological Evaluation, Molecular Docking and Molecular Dynamics of Substituted Thieno[2,3-d]pyrimidine Derivatives as Potential Anti-Alzheimer Agents

Spectral Data of Synthesized Products

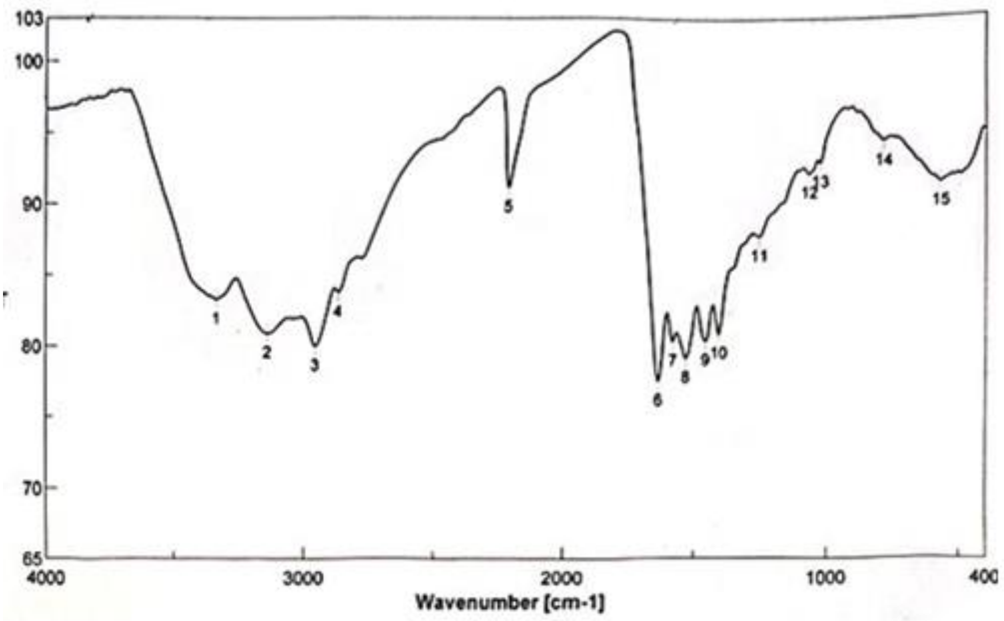

Figure S 1. IR spectrum of compound **1**

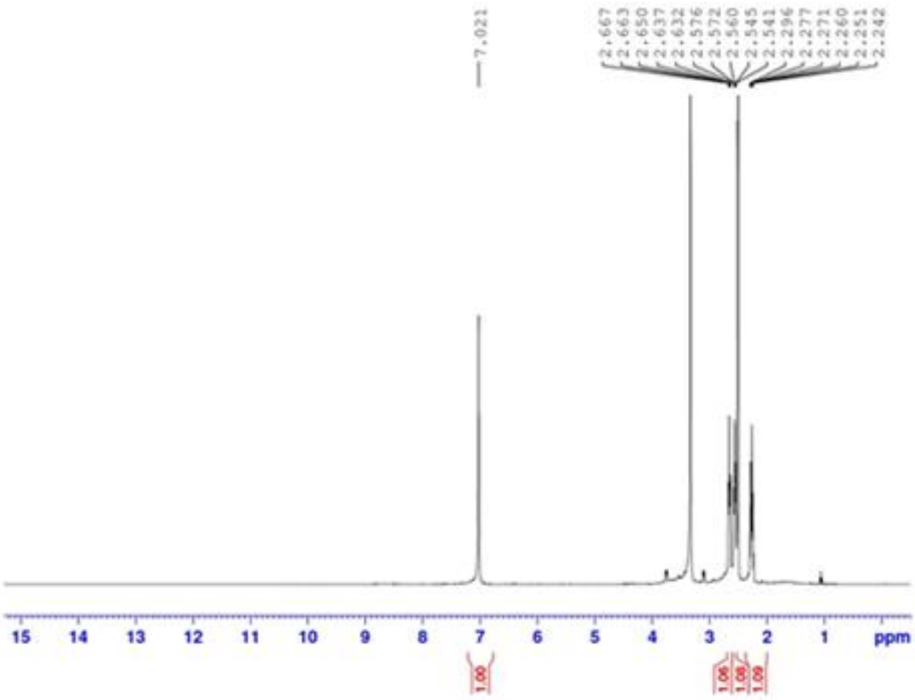

Figure S 2. ^{1}H NMR spectrum of compound **1**

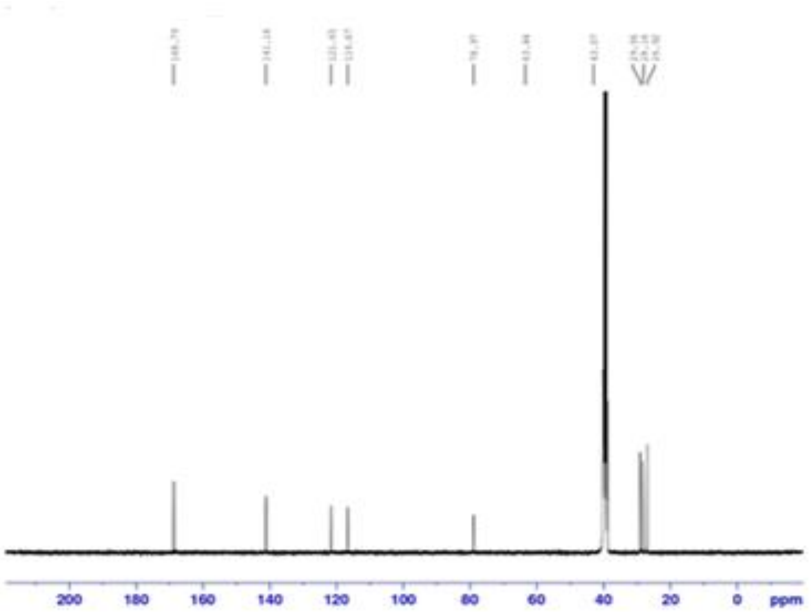

Figure S 3. ^{13}C NMR spectrum of compound **1**

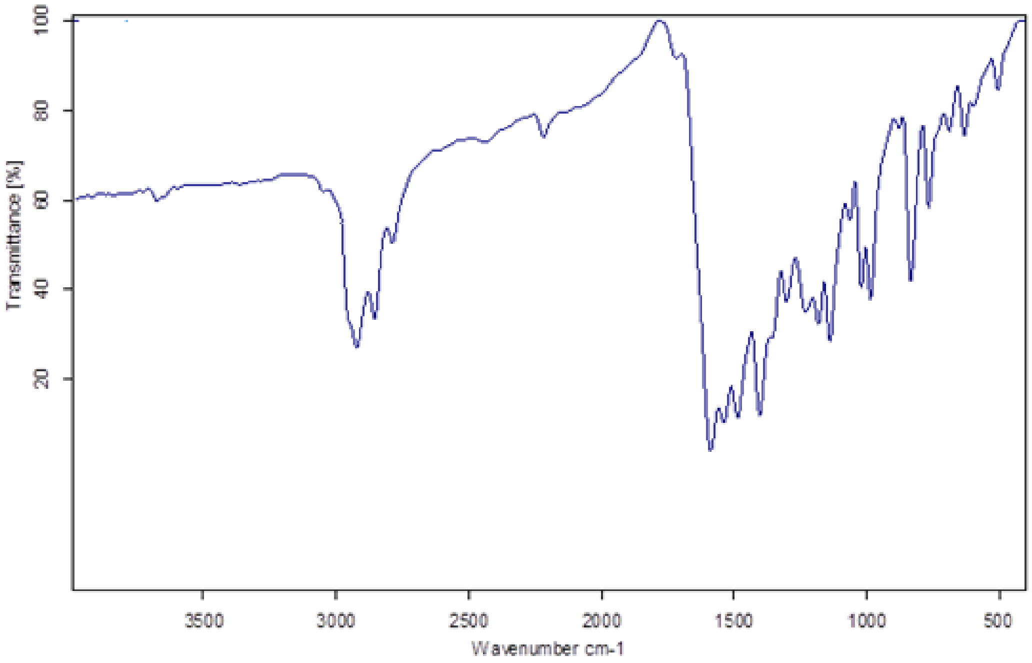

Figure S 4. IR spectrum of compound **2**

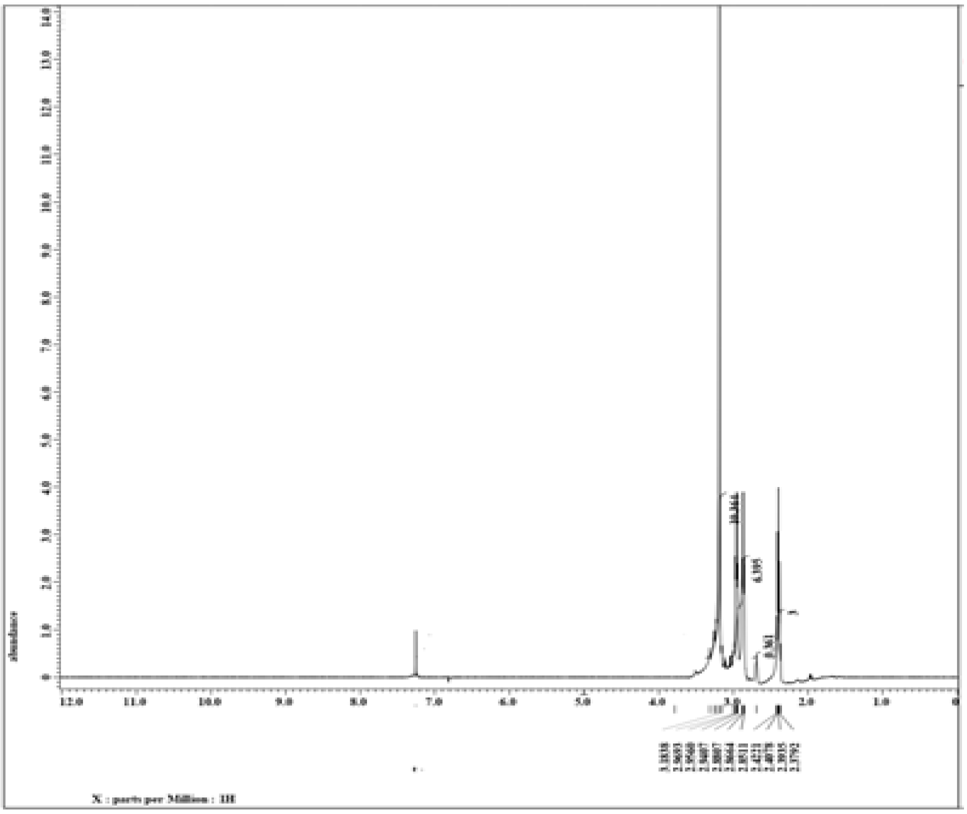

Figure S 5. ^{1}H NMR spectrum of compound **2**

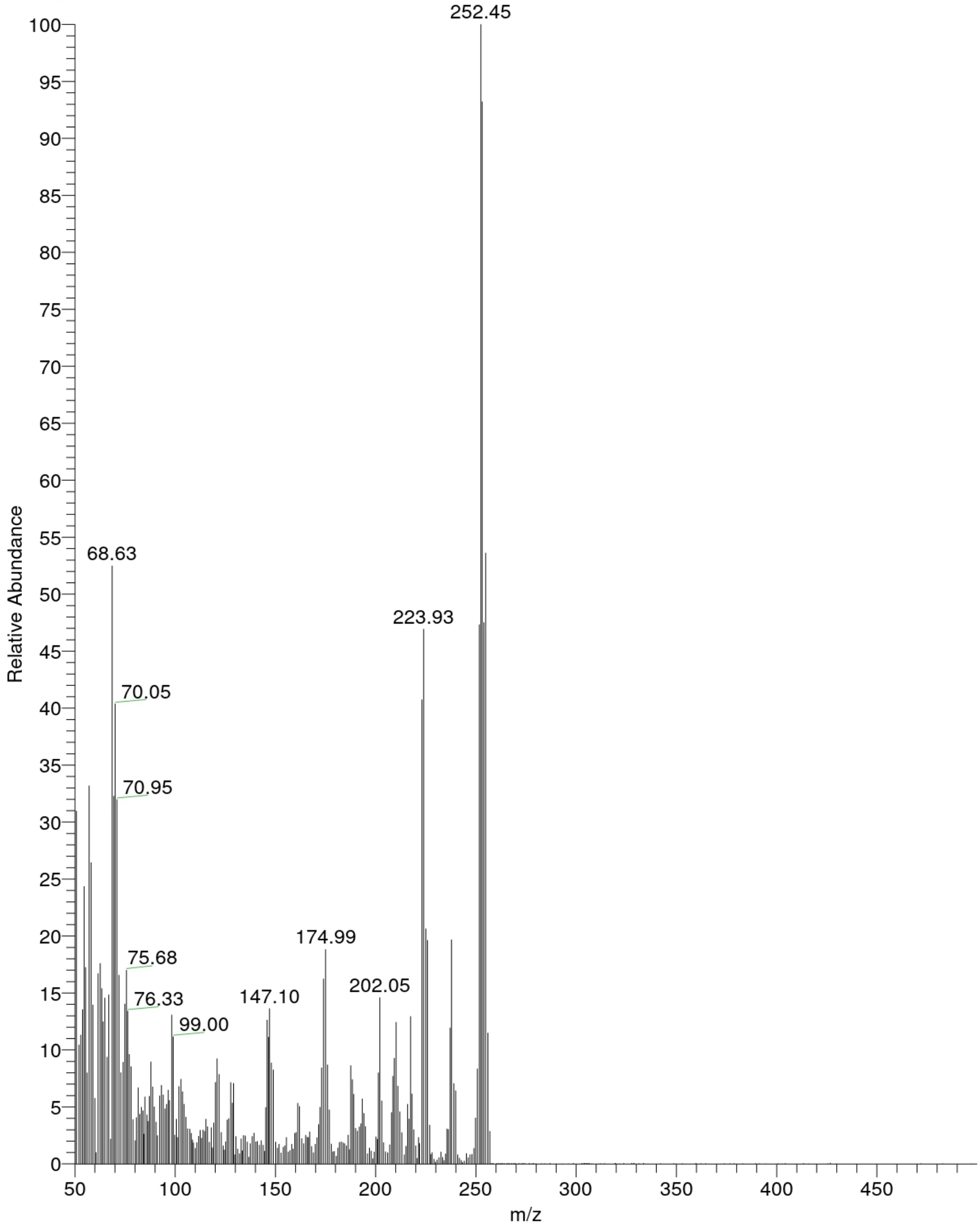

Figure S **6**: Mass spectrum of compound **2**

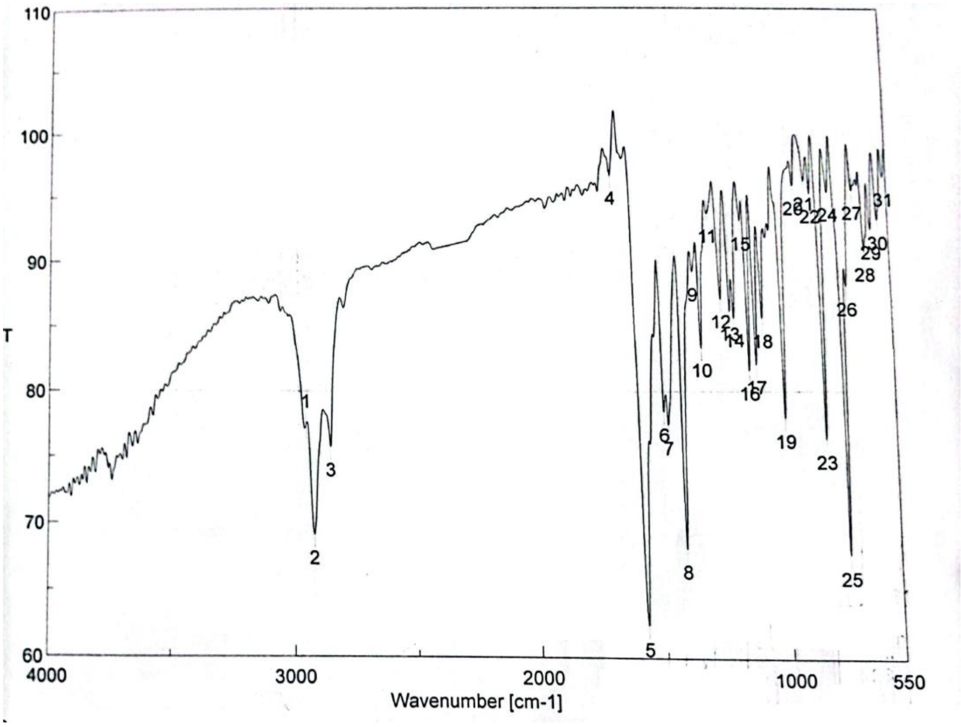

Figure S 7. IR spectrum of compound **3**

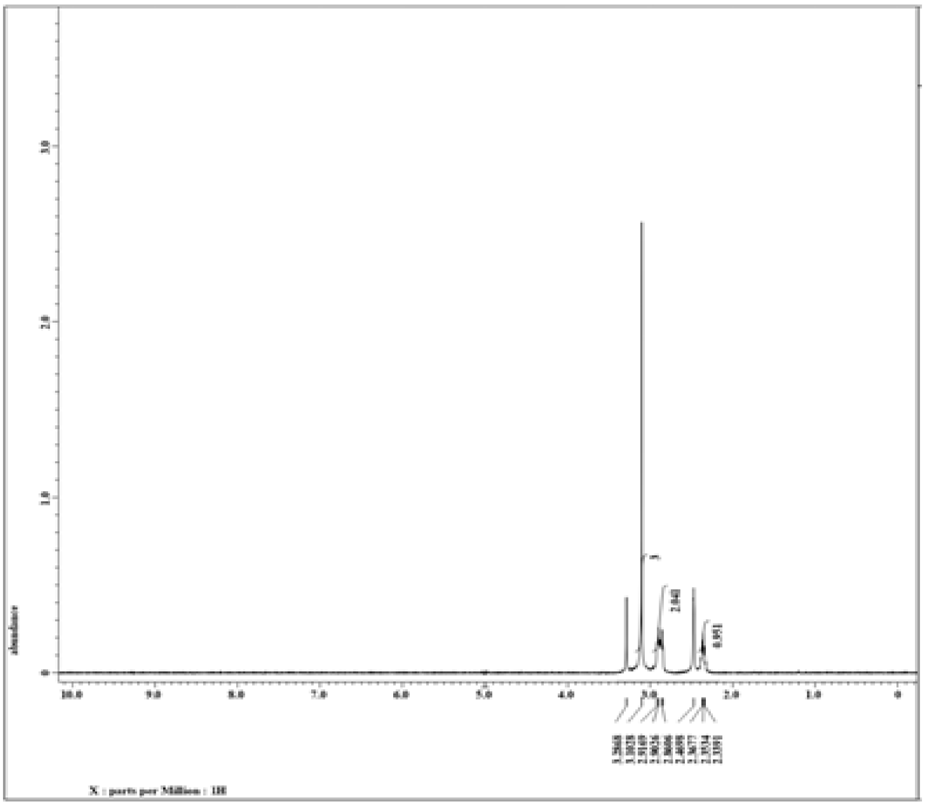

Figure S 8. ^{1}H NMR spectrum of compound **3**

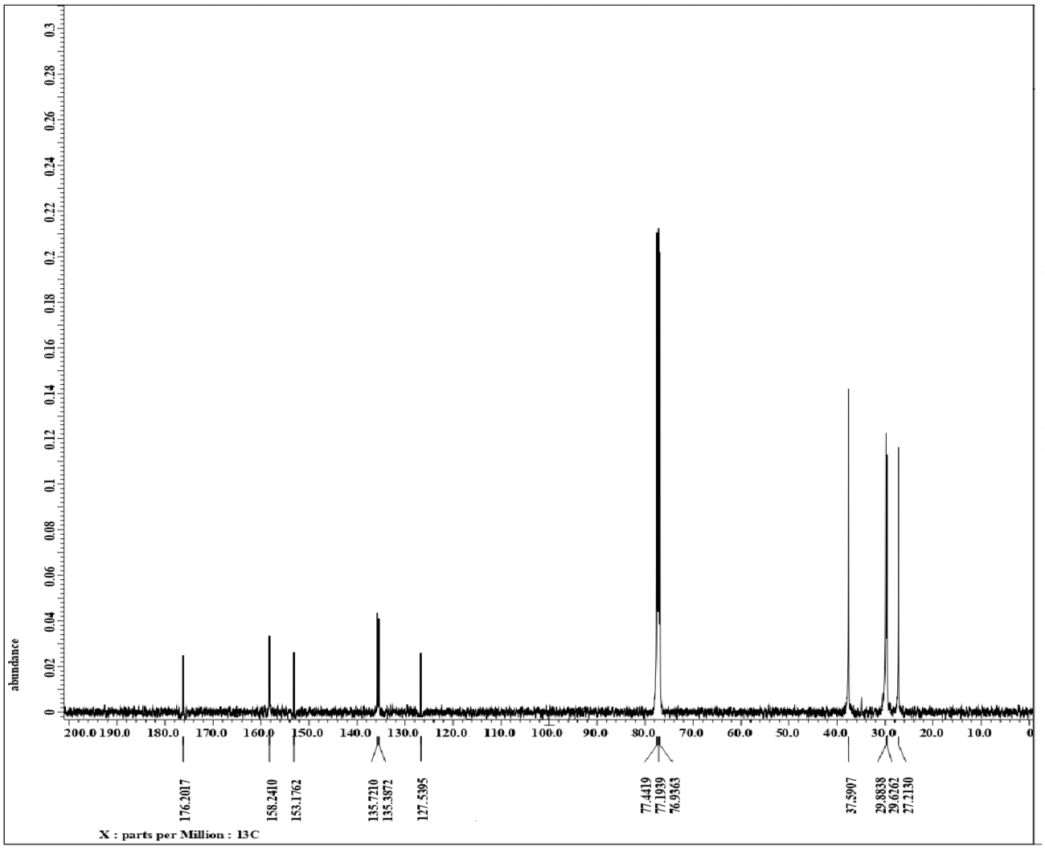

Figure S 9. ^{13}C NMR spectrum of compound **3**

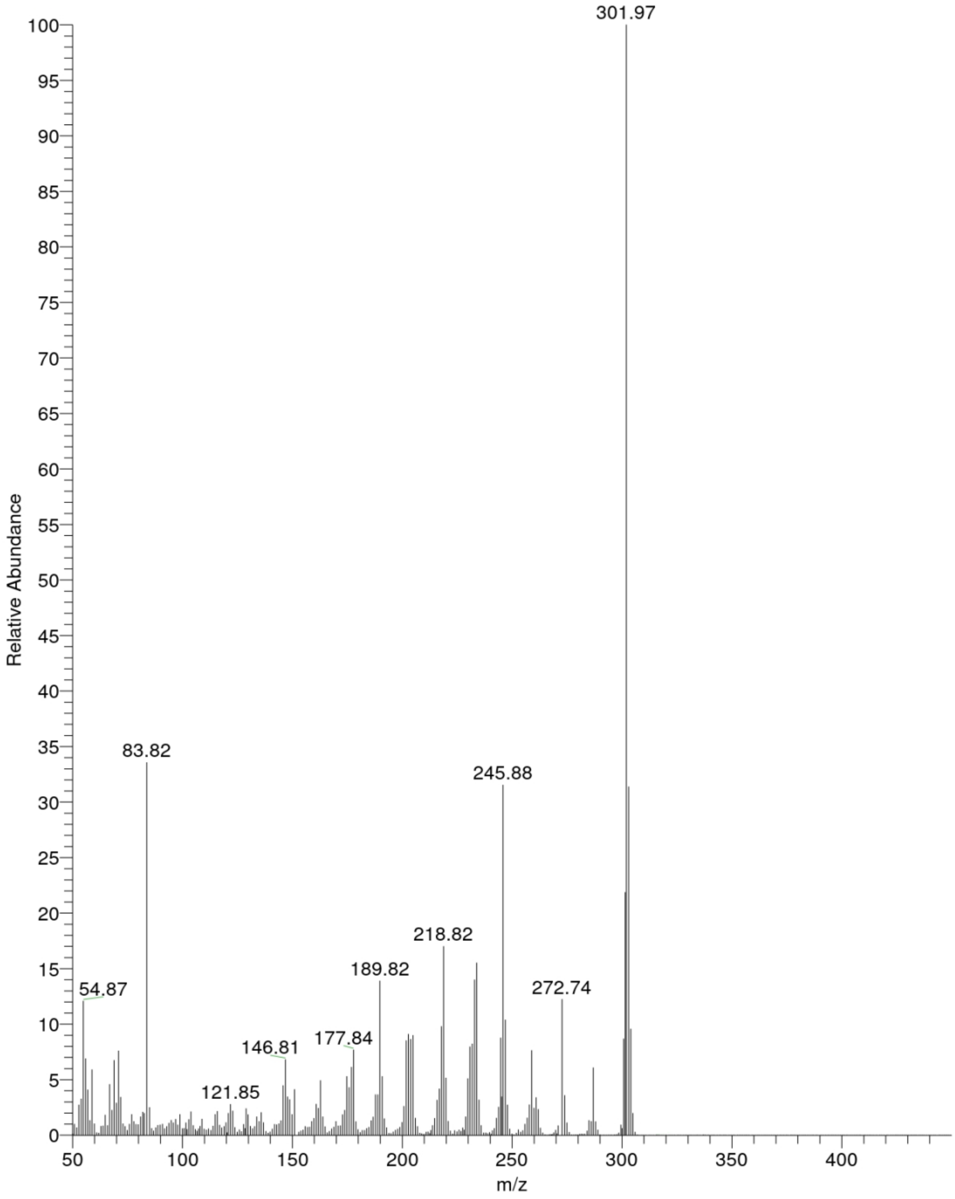

Figure S 10. Mass spectrum of compound **5**

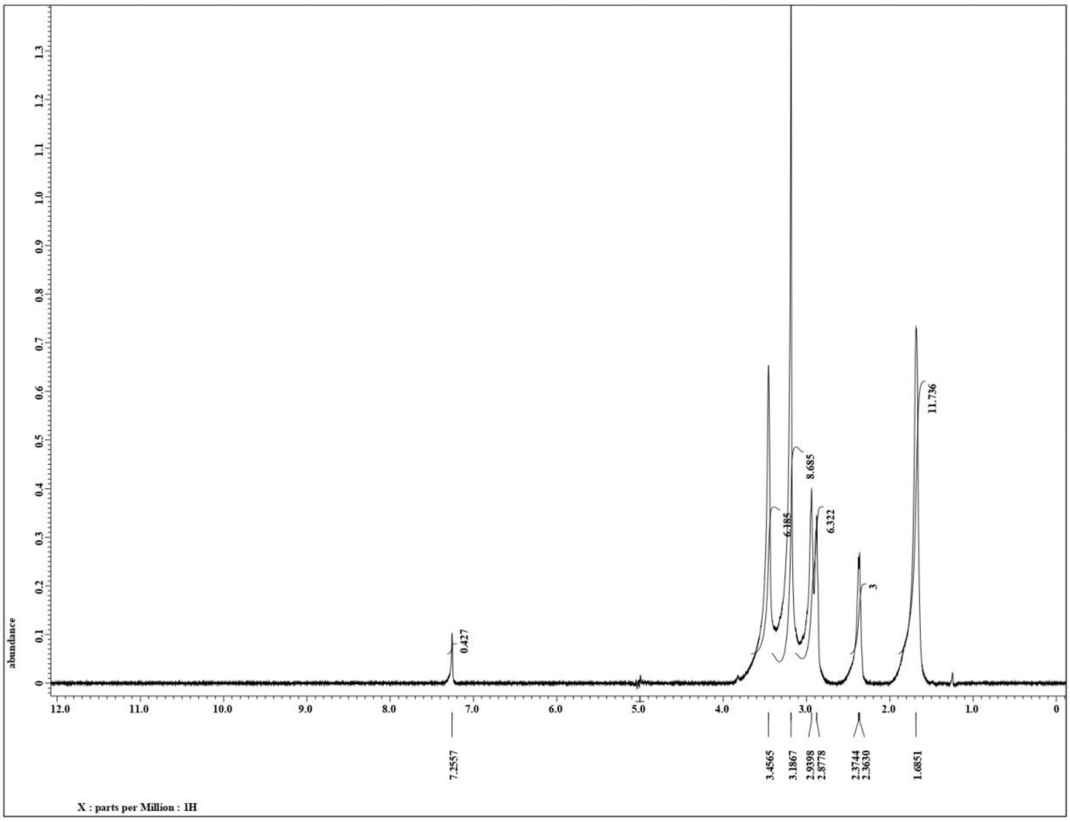

Figure S 11. ^{1}H NMR spectrum of compound **5**

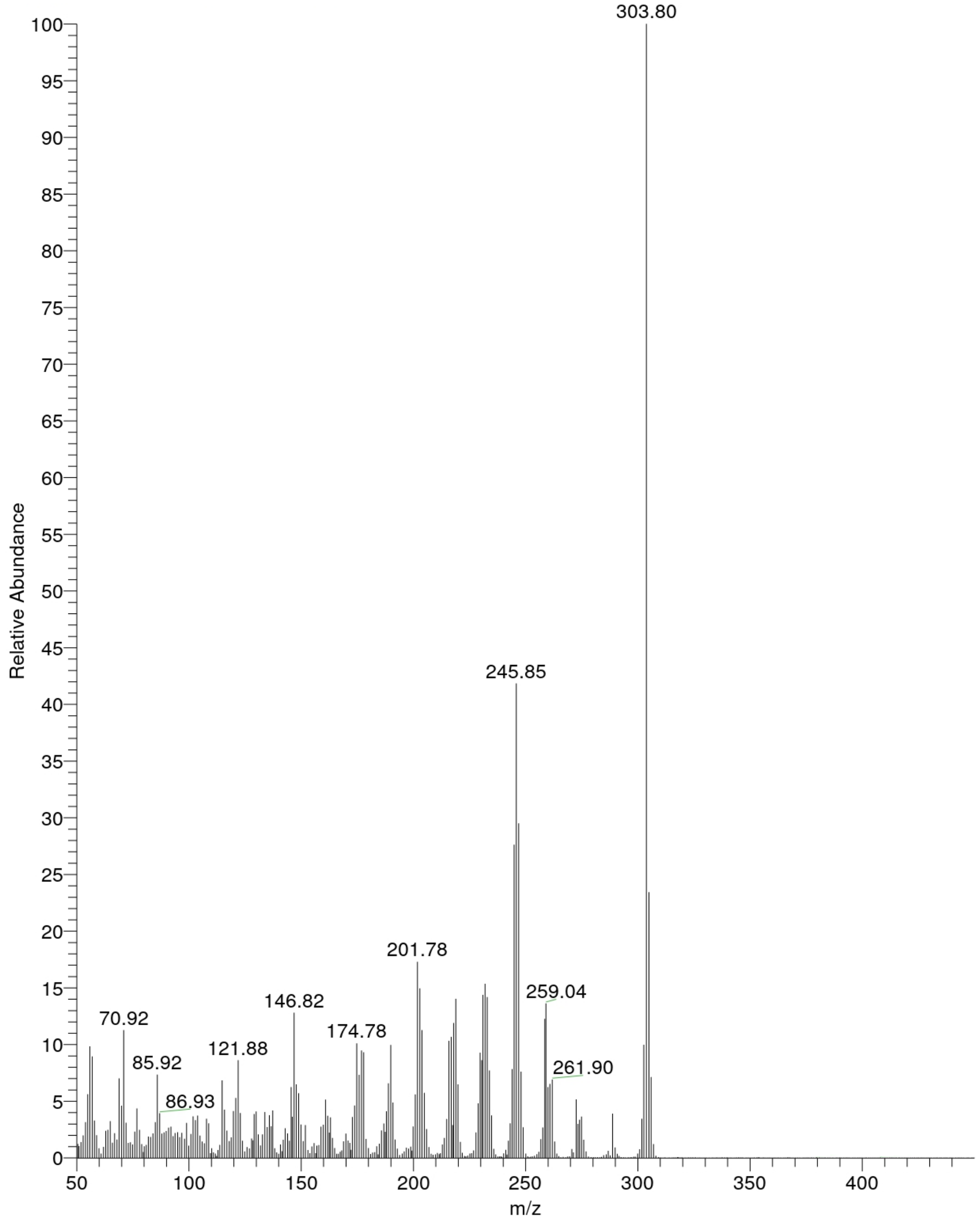

Figure S 12. Mass spectrum of compound **6**

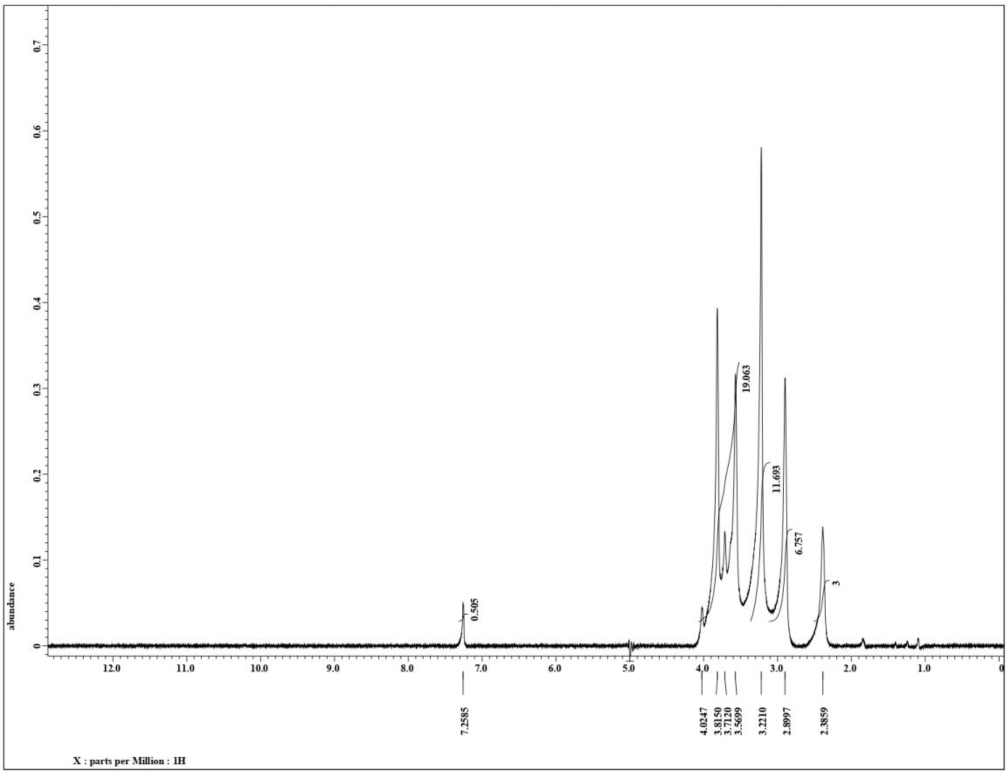

Figure S 13. ^{1}H NMR spectrum of compound **6**

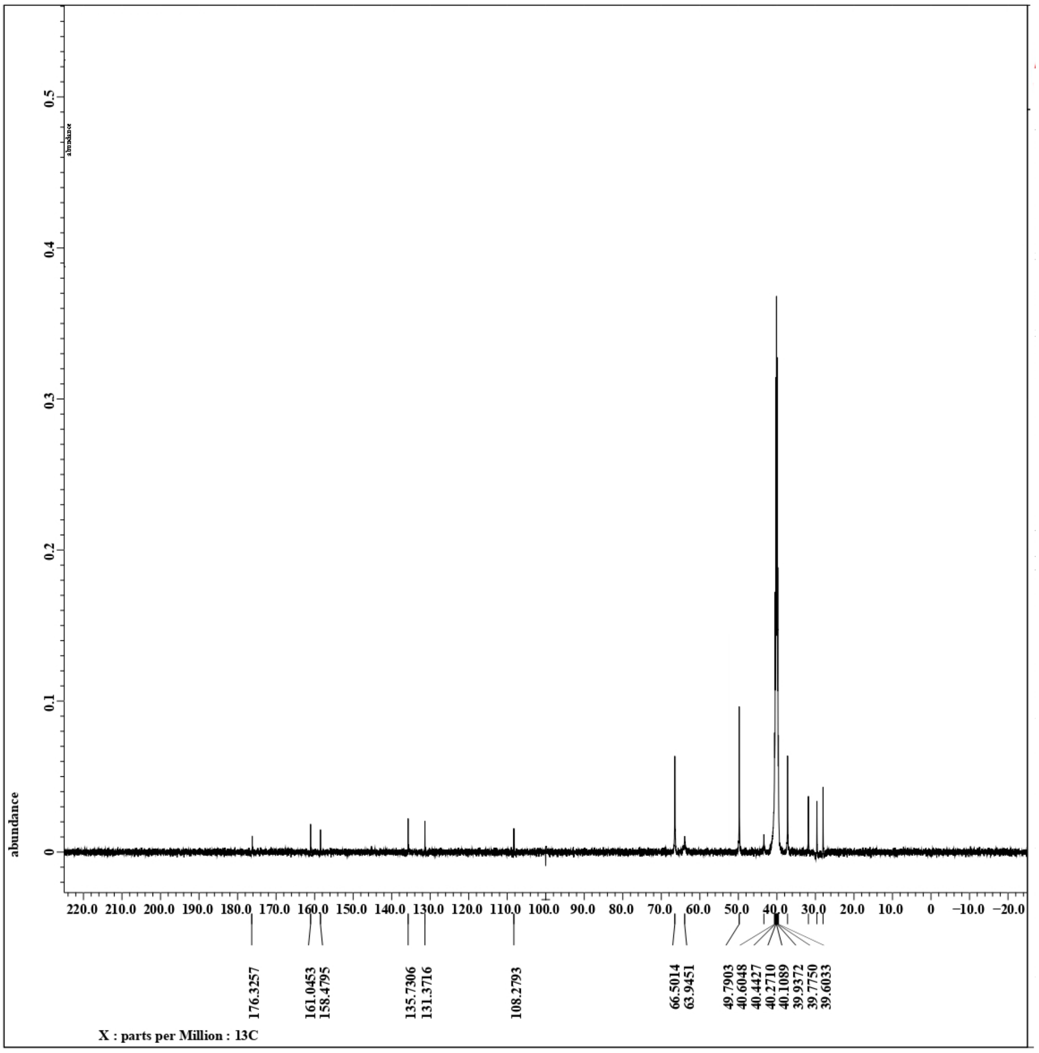

Figure S 14. ^{13}C NMR spectrum of compound **6**

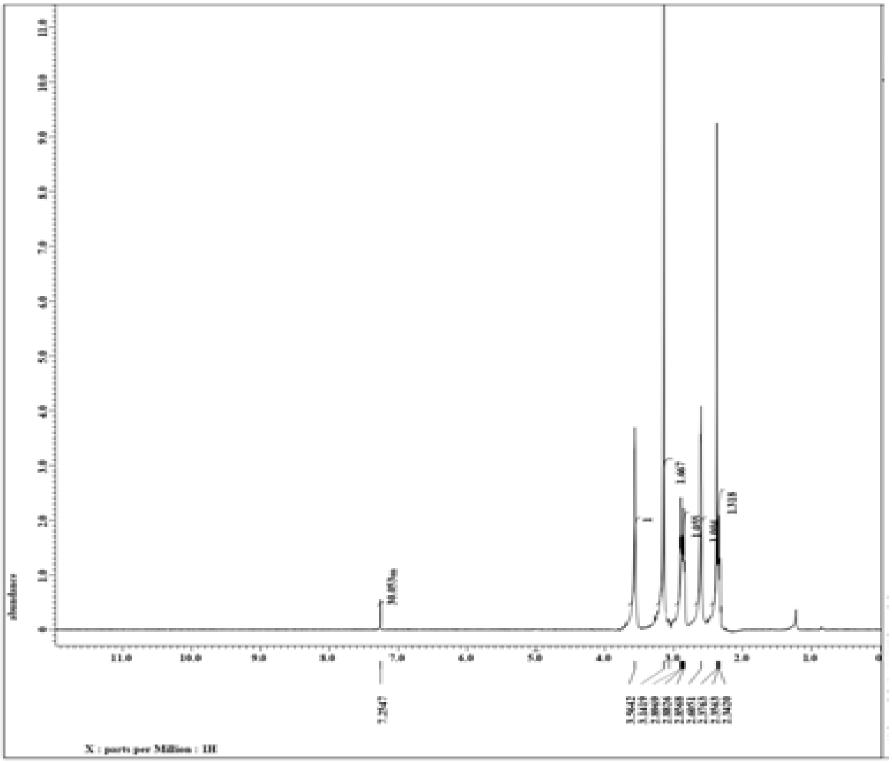

Figure S 15. ^{1}H NMR spectrum of compound **7**

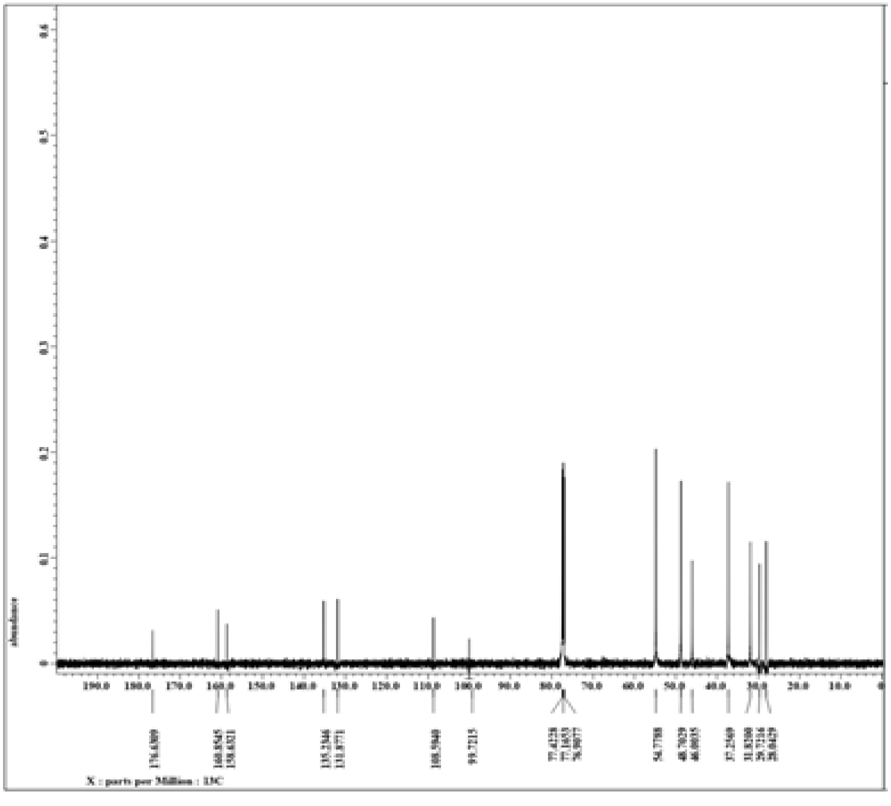

Figure S 16. ^{13}C NMR spectrum of compound **7**

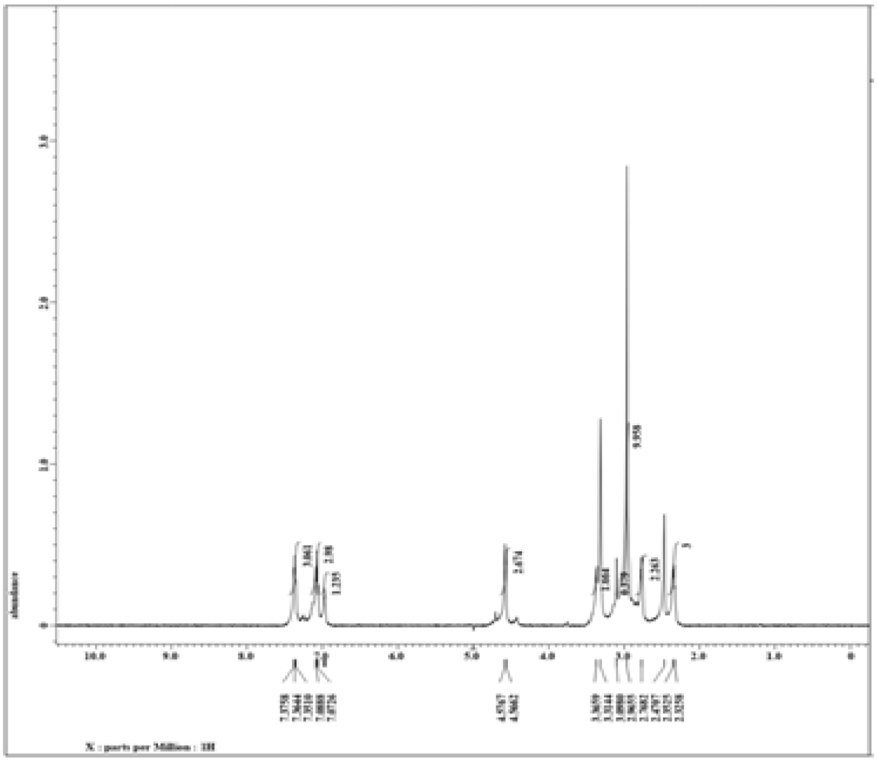

Figure S 17. ^{1}H NMR spectrum of compound **8**

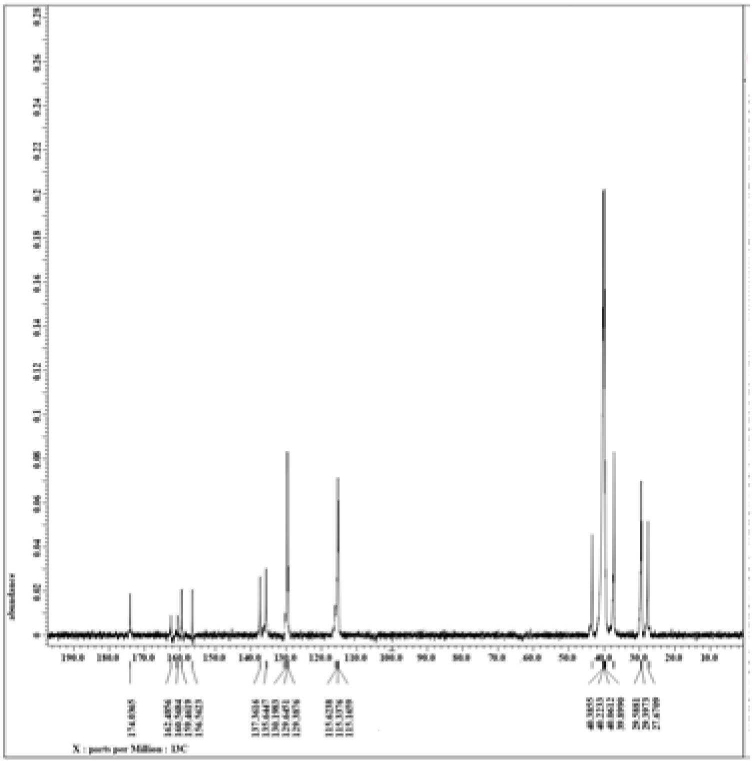

Figure S 18. ^{13}C NMR spectrum of compound **8**

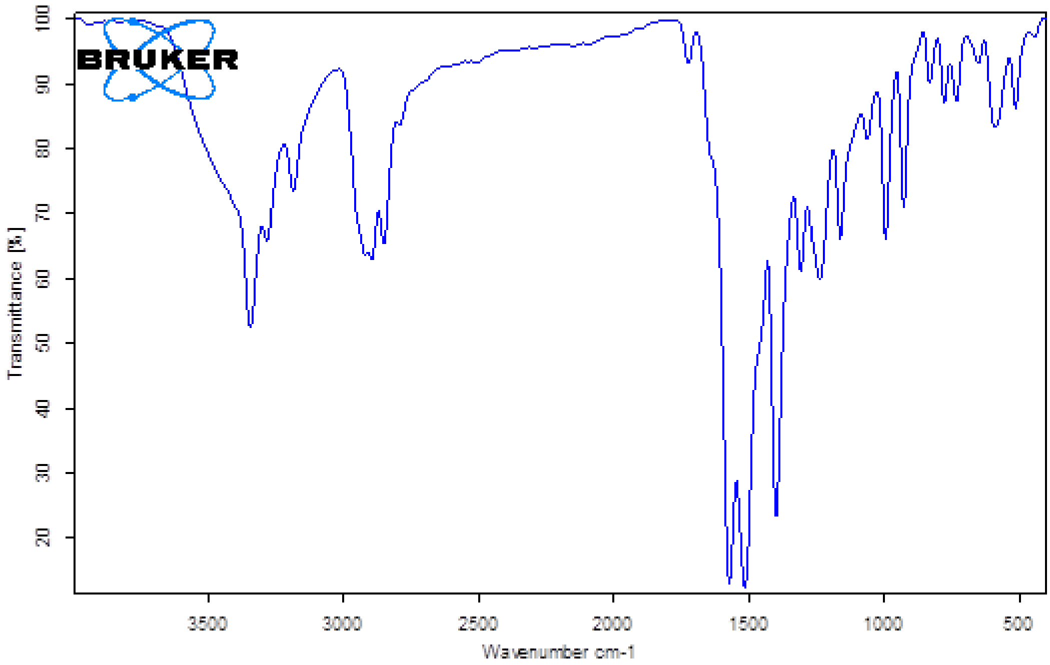

Figure S 19. IR spectrum of compound **9**

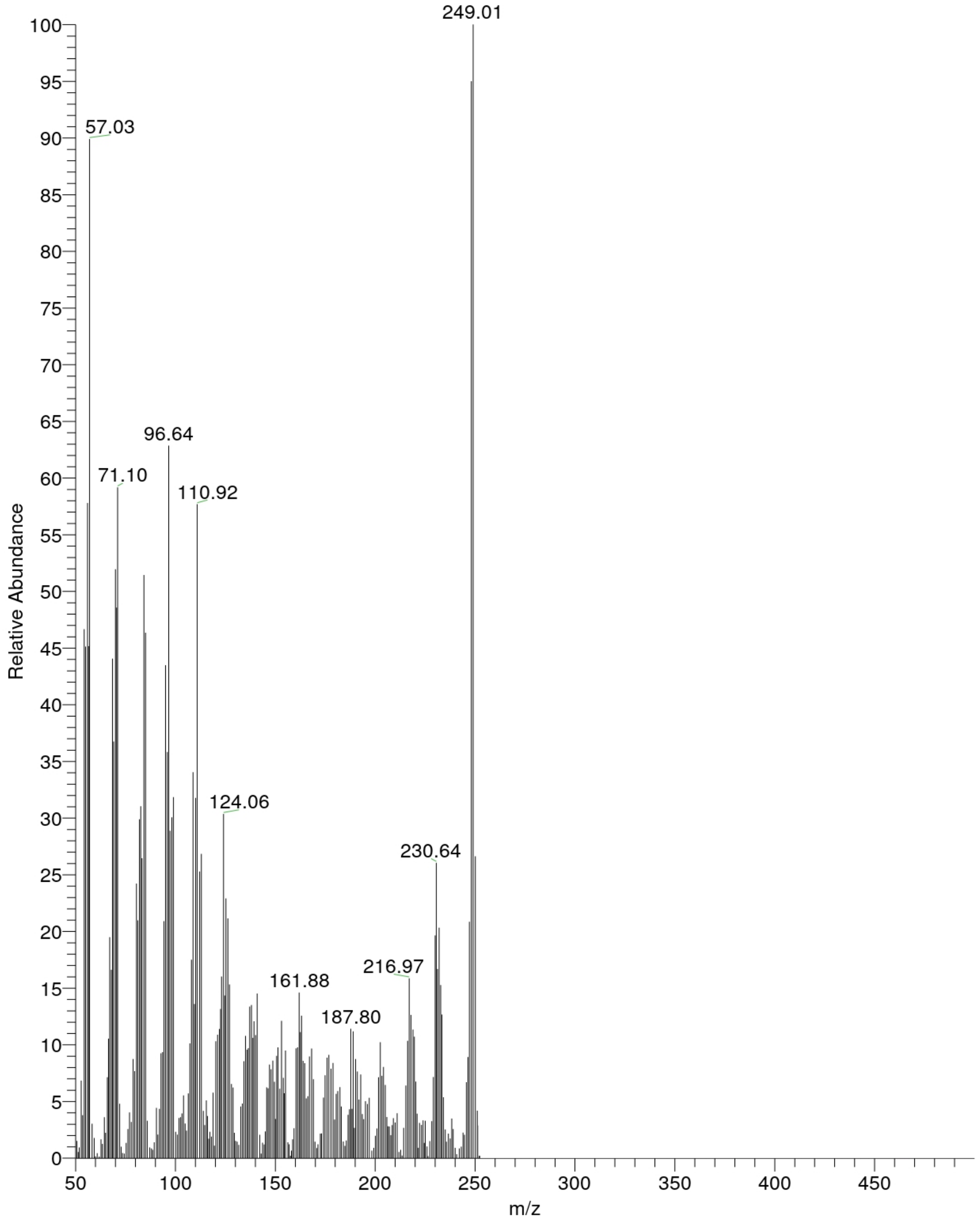

Figure S 20. Mass spectrum of compound **9**

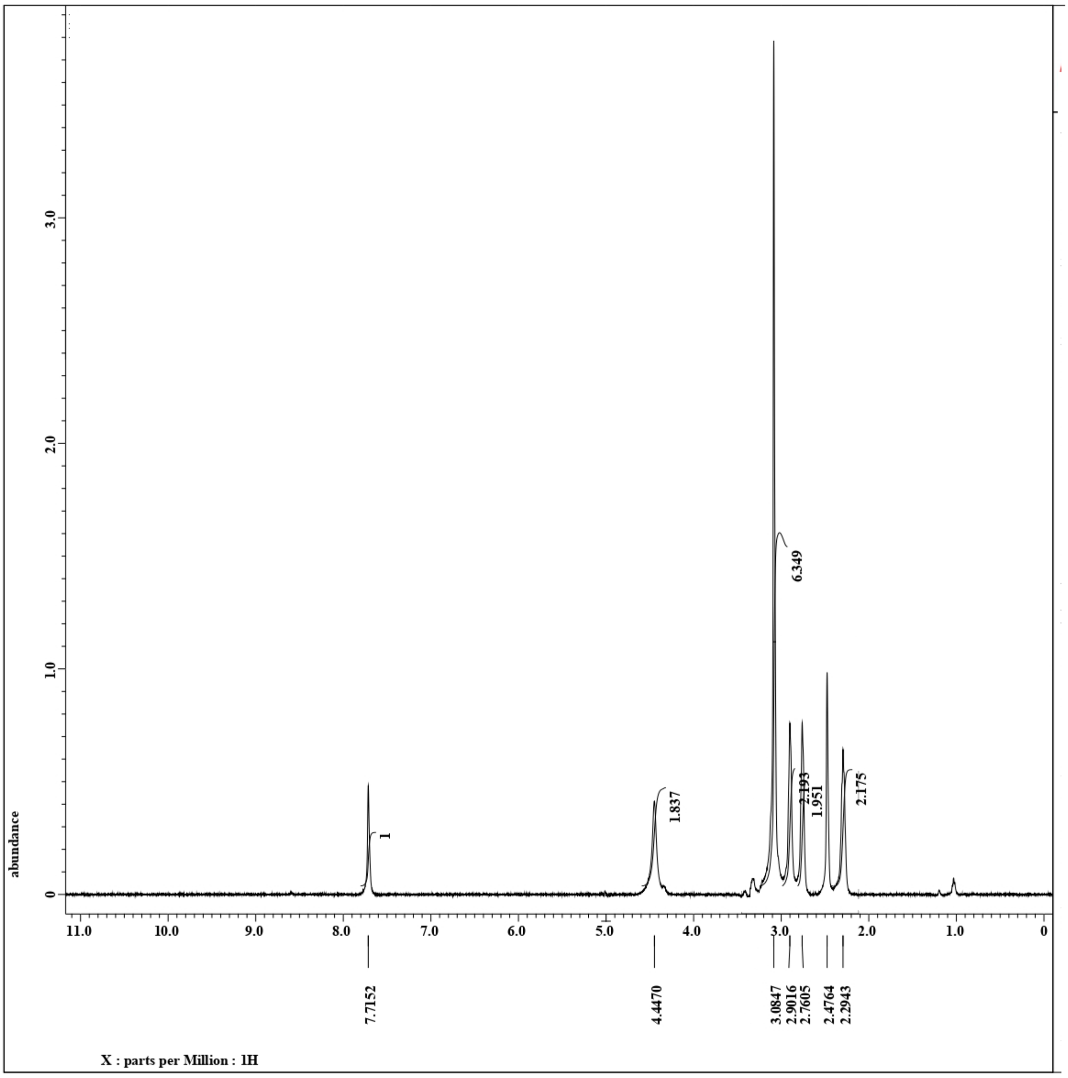

Figure S 21. ^{1}H NMR spectrum of compound **9**

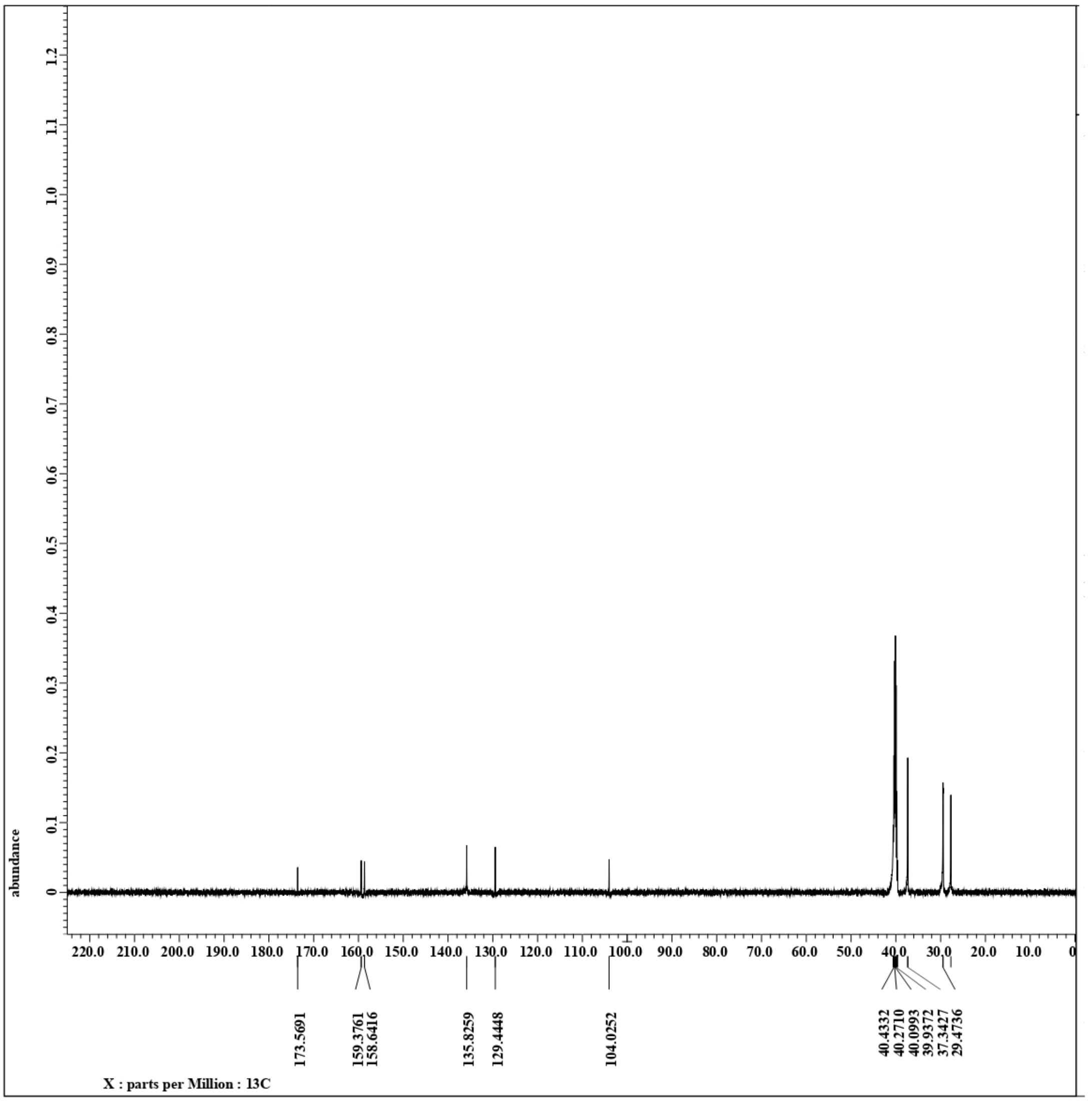

Figure S 22. ^{13}C NMR spectrum of compound **9**

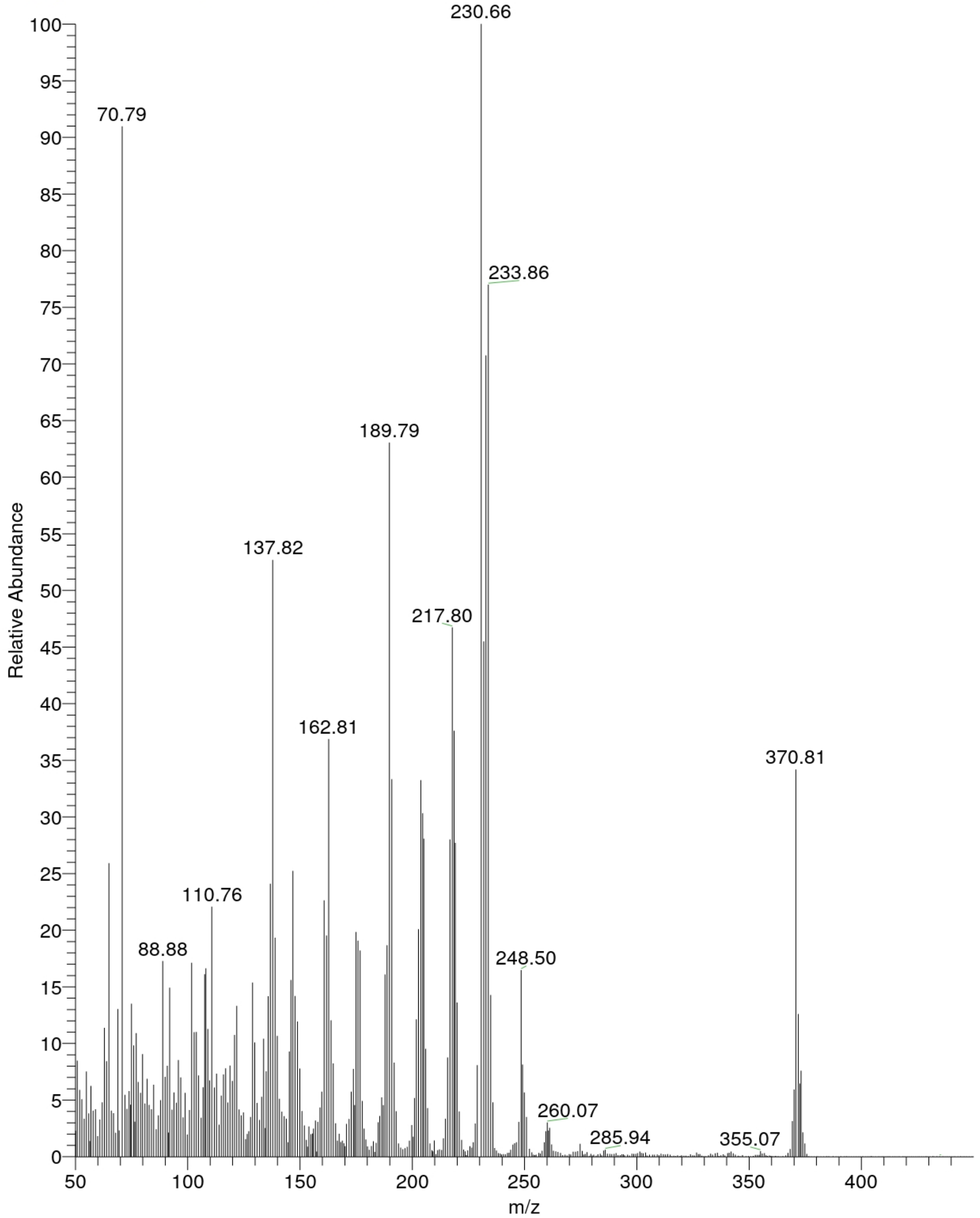

Figure S 23. Mass spectrum of compound **10**

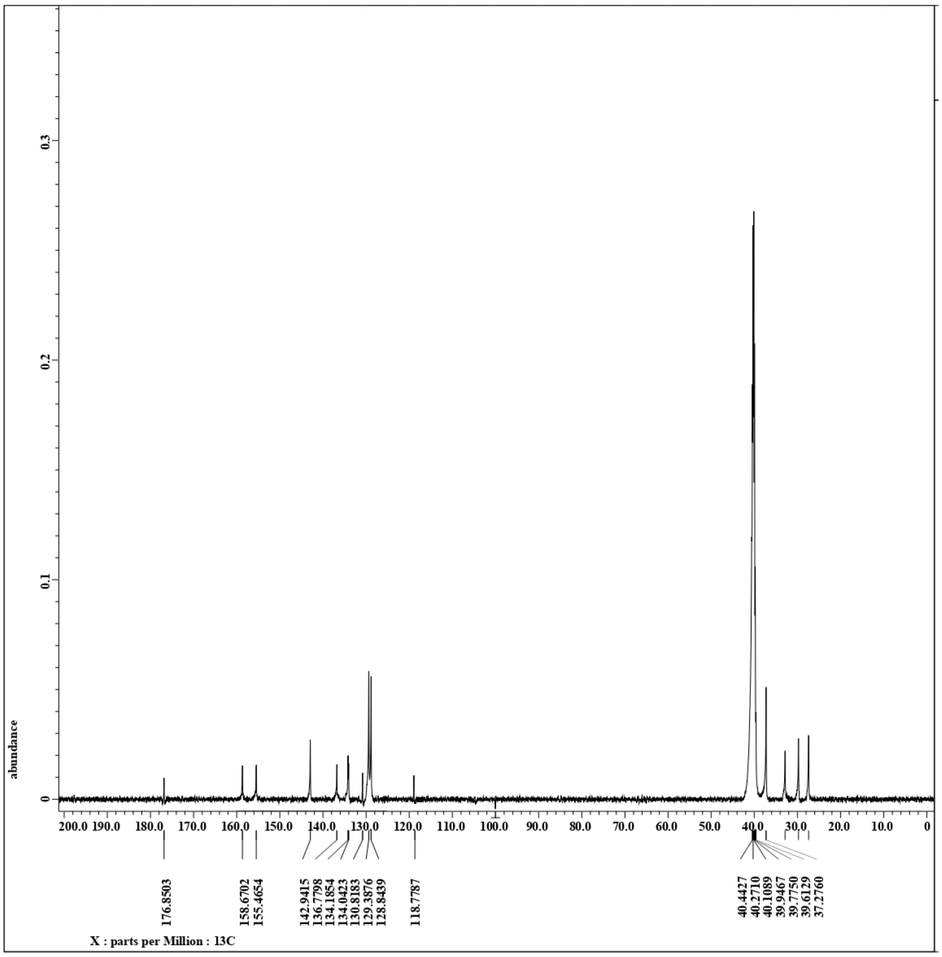

Figure S 24. ^{13}C NMR spectrum of compound **10**

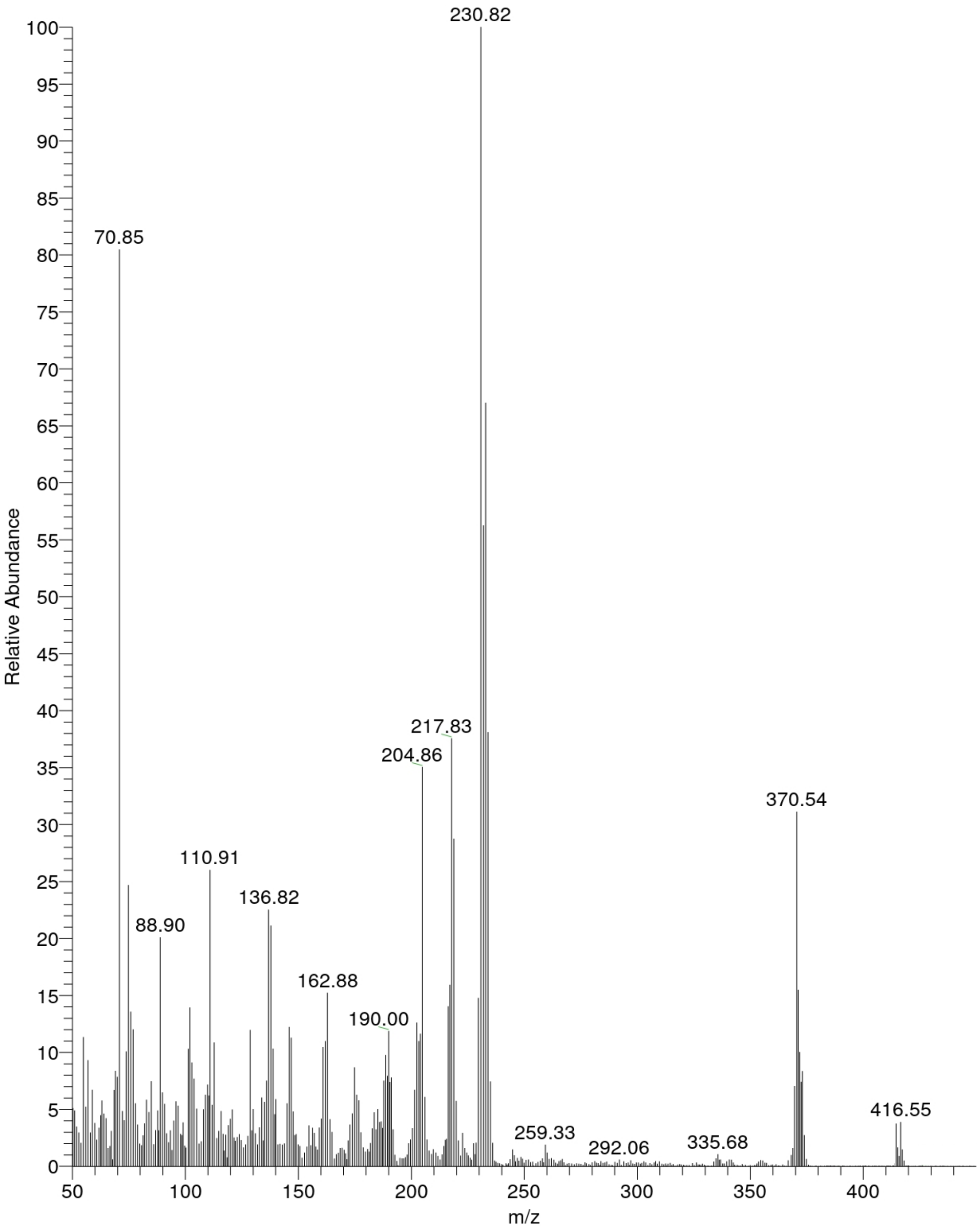

Figure S 25. Mass spectrum of compound **11**

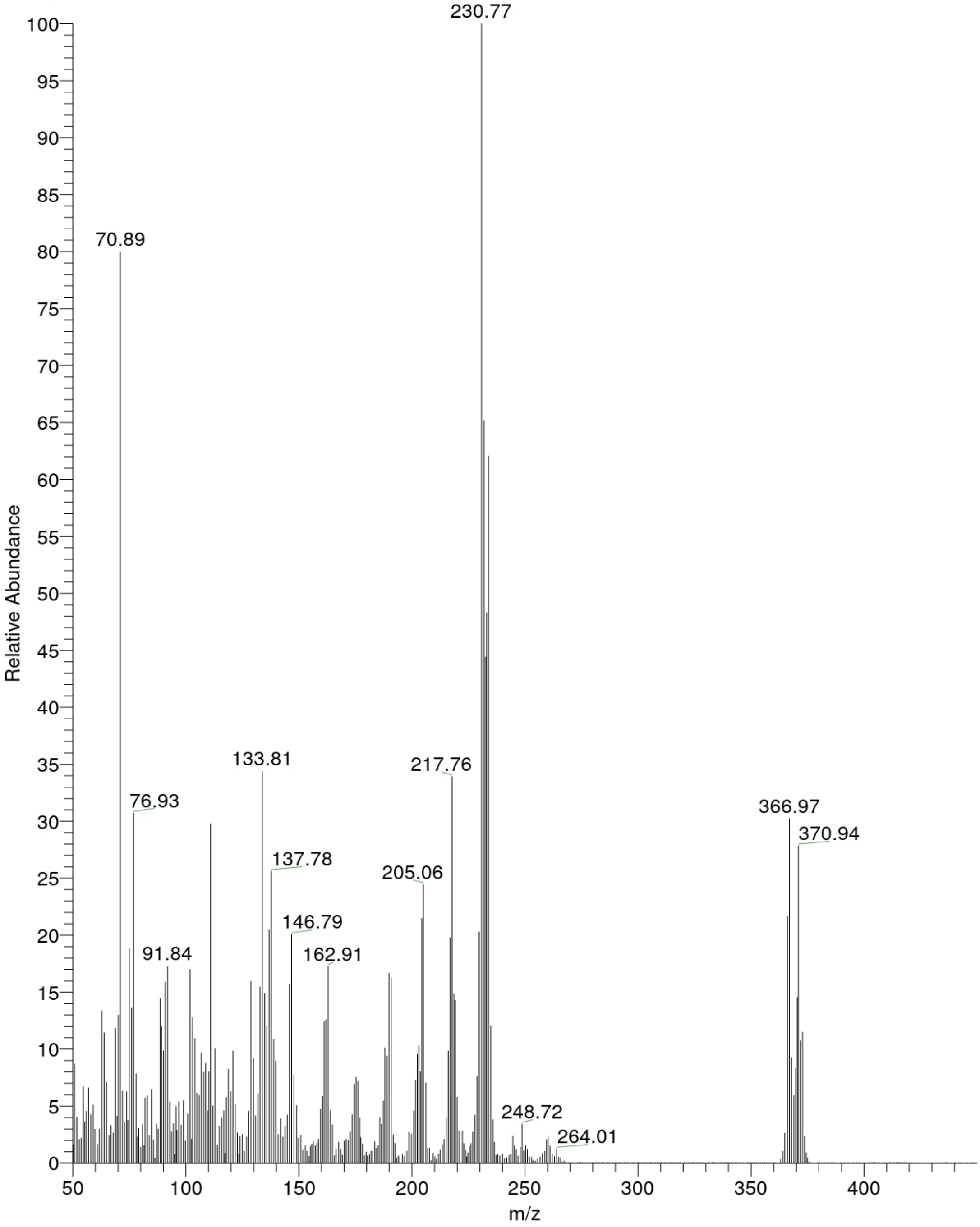

Figure S 26. Mass spectrum of compound **13**

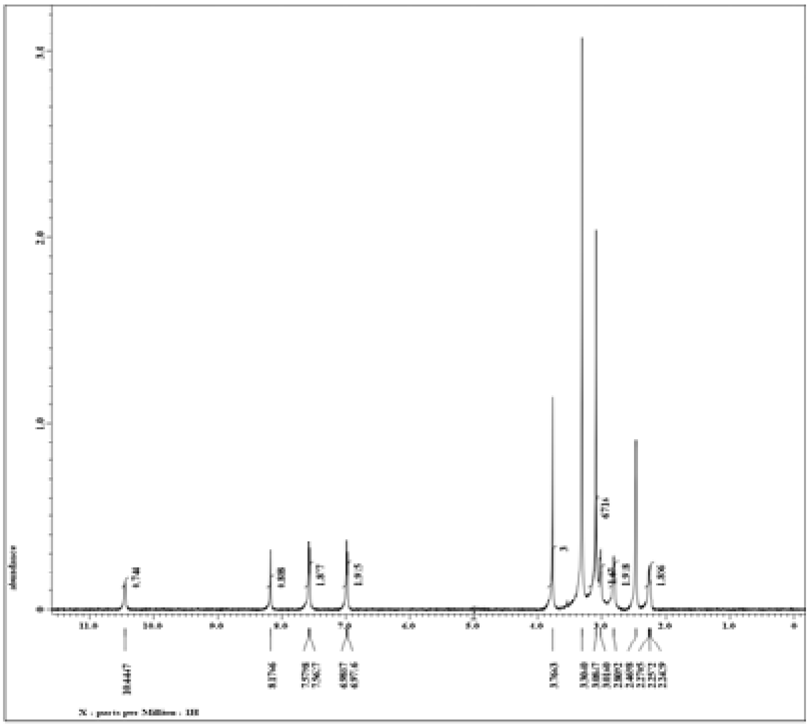

Figure S 27. ^{1}H NMR spectrum of compound **13**

Table S1. Representation of the synthesized compounds (3–13) bound to human acetylcholinesterase receptor

Compounds	Pose	Docking score	Type of Interaction
**3**	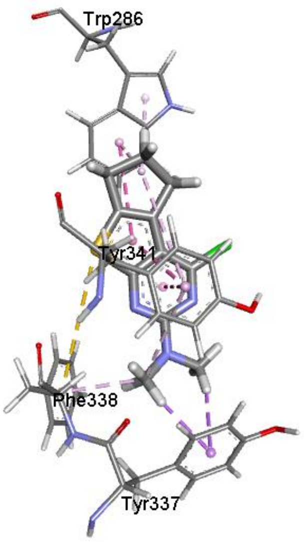	-7.4	Pi-sigma with 337<br>Pi-pi stacking with Tyr 341, Trp 286<br>Pi-sulfur withPhe 338
**4**	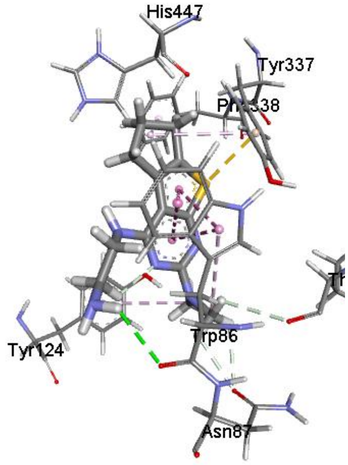	–9.8	Trp 86 from H-bonding , Pi-Pi stacking<br>-pi-sulfur with Tyr 337
**5**	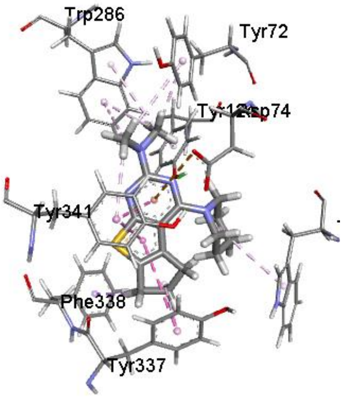	–8.2	-H bonding with Tyr 124<br>Pi-pi stacking with Tyr 341,anf Tyr 337<br>Pi-sigma interaction with Phe338

**6**	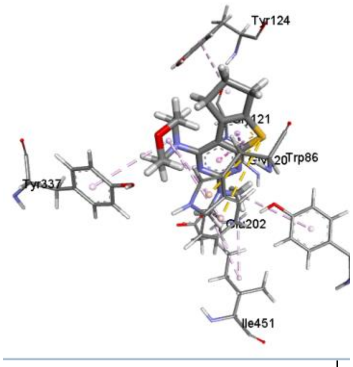	−9.4	Pi-alkyl interaction with Tyr 124, Tyr 337<br>Trp 86 from Pi-sigama , Pi-sulfur
**7**	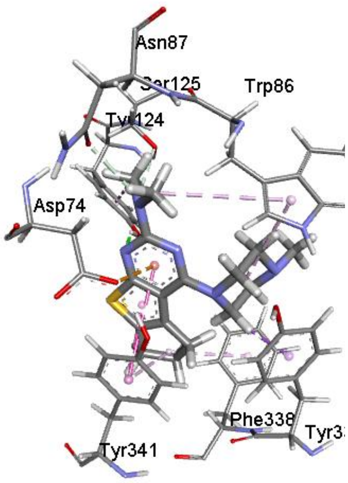	−7.9	H-bond with Tyr 124<br>Pi-anion with Asp 74<br>Pi-pi stacking with Tyr 341
**8**	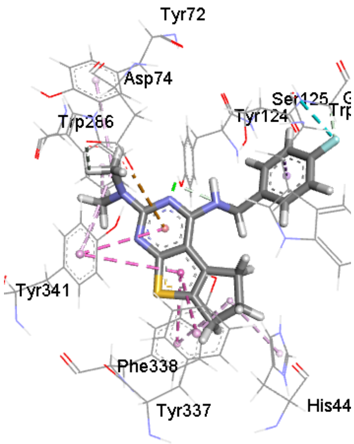	−8.5	Pi-alkyl with Tyr 341 ,Phe 338, Tyr 337<br>H-bond with Tyr 124<br>Pi-anion with Asp 74

**9**	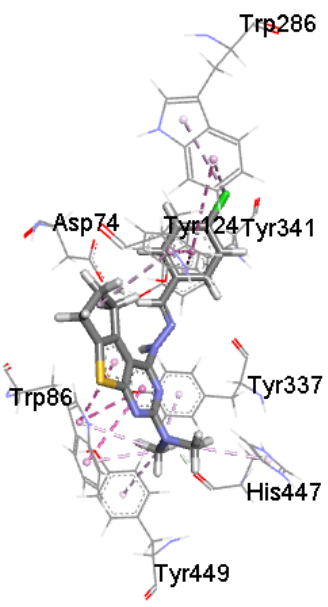	−8.7	PI-Pi stacking with Trp 286<br>H-bond with Phe 295<br>Pi-pi stacking ,and Pi cation with Tyr 341<br>Pi=pi stacking and pi cation interaction with Tyr 341
**10**	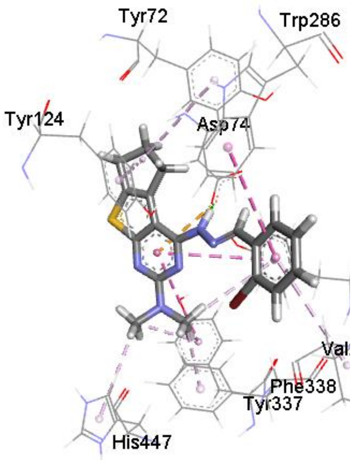	−7.1	Pi-pi stacking with Trp 286, Tyr 341,Tyr 124<br>Pi-alkyl ,and Pi-pi stacking with Trp 86

**11**	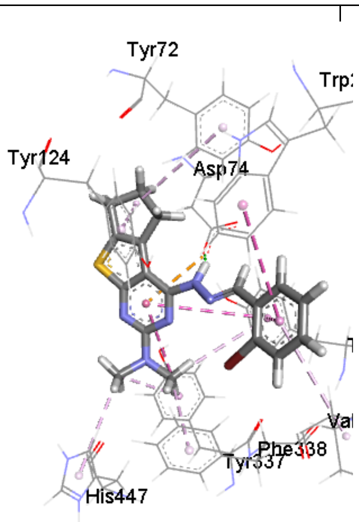	-8.1	Pi-anion , and H-bonding with Asp 74<br>Pi-alkyl interaction with Tyr 337<br>Pi-pi stacking with Trp 286, Tyr 337
**12**	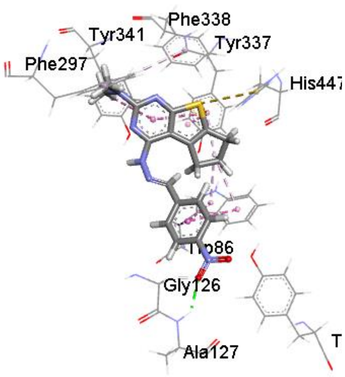	-6.8	Hydrogen bond with Ala 127<br>Pi-PI stacking with Tyr 341<br>Pi-sulfur with His 447 ,Tyr 337<br>Pi-sigma with Trp 86
**13**	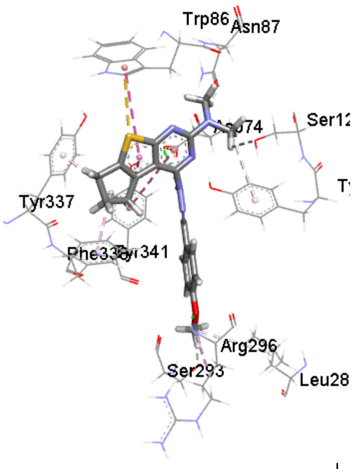	-6.2	H-bond ,and Pi-alkyl interaction with Arg 296<br>H-bond , Pi-anion with Asp 74<br>Pi-pi stacking , and Pi-alkyl interaction with Tyr 341<br>Pi-Pi alkyl interaction with Phe 356

**Donepezil**	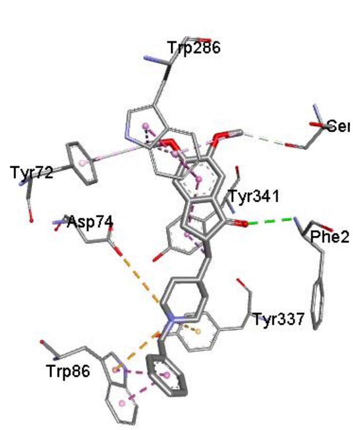	-10.2	-H-bonding interaction with Phe 295<br>-Pi-cation interaction with Trp 86, Asp 74,Tyr 337<br>-pi-sigma wit Tyr 341, Trp 286
**Rivastigmine**	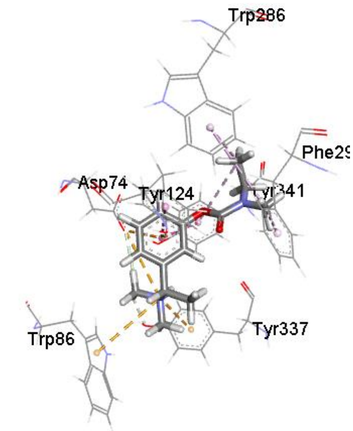	-7.8	-pi-anion with Asp 74,and Trp 86<br>Pi-pi stacking with Tyr 341<br>Pi-lone pair interaction with Tyr 124

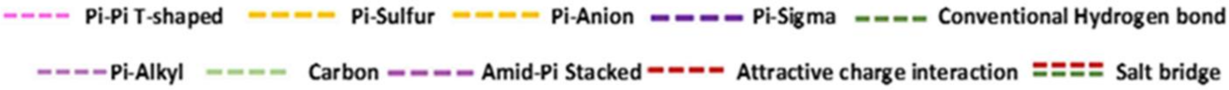

a

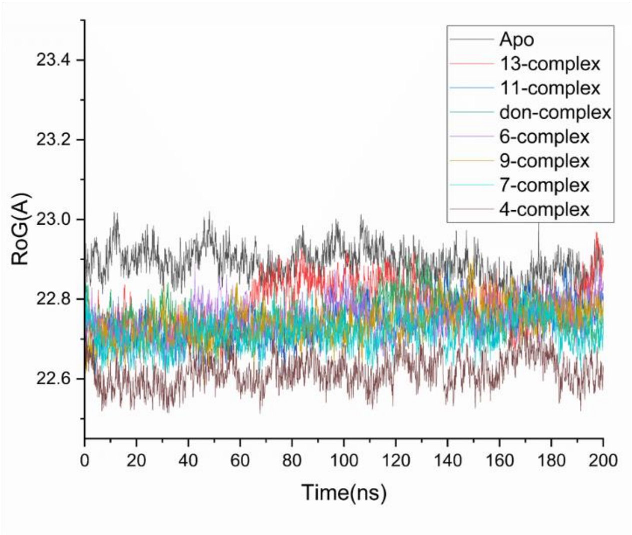

b

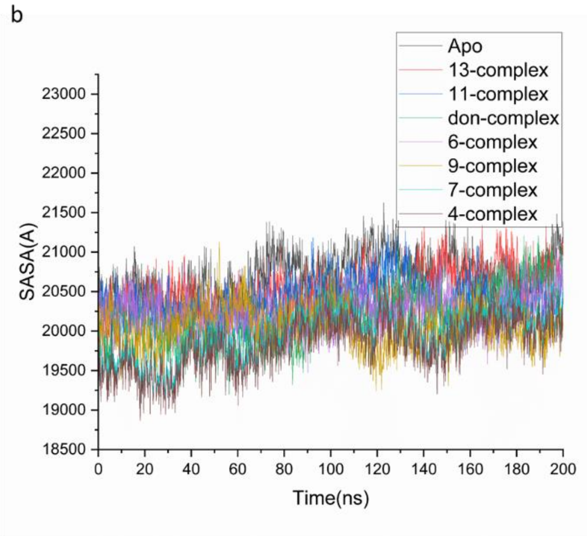

Figure S28: (a) ROG of Cα atoms of protein residues; (b) solvent accessible surface area (SASA) of the C α of the backbone atoms relative (black) to the starting minimized over 200 ns for the catalytic binding site of human acetylcholinesterase receptor with compound **13**(red), compound **11** (blue), Donepezil(green), compound 6 (classical blue), compound **9** (pink), compound 9 (blue),compound 4 (brown) .
